# Experimental designs used for optimising the effects of health interventions and implementation strategies: a scoping review

**DOI:** 10.1186/s12913-025-13184-9

**Published:** 2025-08-25

**Authors:** Erin Nolan, Luke Wolfenden, Taylor Benn, Elizabeth Holliday, Daniel Barker, Christopher Oldmeadow, Alix Hall

**Affiliations:** 1 School of Medicine and Public Health, The University of Newcastle, Callaghan, NSW Australia; 2 Hunter Medical Research Institute, New Lambton Heights, NSW Australia; 3 National Centre of Implementation Science, The University of Newcastle, Wallsend, NSW Australia; 4 Hunter New England Population Health, Hunter New England Area Health Service, Wallsend, NSW Australia

**Keywords:** Implementation, Optimisation, Study design, Trial design

## Abstract

**Background:**

Optimisation is the iterative process to improve a health intervention or implementation strategy within resource constraints. This review aimed to identify which study designs are being used to evaluate the optimisation of health interventions and implementation strategies, and whether they differ by optimisation target. This review identifies possible strategies to improve future optimisation trials.

**Methods:**

A scoping review of the Medline, EMBASE, CINAHL, and ProQuest Nursing and Allied Health Source databases was undertaken. The International Clinical Trials Registry Platform and the Australian New Zealand Clinical Trials Registry were also searched for relevant trials. Data were extracted by one reviewer for 64% of studies, and by two reviewers for 36% of studies. Data extracted included research designs, optimisation target and constraints, and whether an optimisation framework and criteria for optimisation success was used. The frequency of optimisation constraints was tabulated by experimental design and optimisation target.

**Results:**

183 studies aimed to optimise an intervention (*n* = 142) or implementation strategy (*n* = 39) or both (*n* = 2). Factorial designs were the most common design used to evaluate optimisation of an intervention (41%), whereas pre-post designs were the most common for implementation strategies (46%). Optimisation success was defined in 11% of trials and 24% of trials used a framework for optimisation.

**Conclusions:**

This review characterises the design features of trials aiming to optimise health interventions or implementation strategies. There is a need for the use of frameworks to guide trial design and for a clear definition of optimisation success. It is recommended to consider using alternate methods that may overcome common impediments and align better with optimisation, such as adaptive designs and Bayesian statistics.

**Supplementary Information:**

The online version contains supplementary material available at 10.1186/s12913-025-13184-9.

## Contributions to the literature

- Optimisation is a proposed approach to overcome the shortcomings of current intervention and implementation trials.
- Our study identified that trials aiming to optimise a health intervention or implementation strategy rarely use optimisation frameworks or have a clear definition of optimisation success.
- We recommend the use of optimisation frameworks and a clear definition of optimisation success, as well as consideration of alternate methods such as adaptive designs, Bayesian statistics, and consolidating samples across research groups to overcome the impediments to evaluating optimisation success.

## Background

Significant public funding is invested in the development and implementation of interventions to improve patient and community health [1]. A criticism of the current research production process, however, is that promising health innovations may be prematurely abandoned following a pilot or early stage trial, or that those found to be efficacious when tested under ideal research conditions [2] fail to yield benefits when applied in a real-world setting, often due to poor implementation [3]. To improve the impact of investments in health research, the development of effective and ‘implementable’ interventions, and the development and testing of strategies capable of implementing with sufficient fidelity to accrue benefit is needed.

How amenable interventions are to implementation in more real-world contexts is often not sufficiently considered when developing new interventions. Many interventions tested in efficacy trials, for example, require resources, expertise or infrastructure to deliver that are not readily available in the settings they are ultimately intended to be implemented [3, 4]. Such interventions require significant adaptation in order to be feasibly and acceptably integrated into routine practice [3, 4]. It has also been suggested that there is an over investment by research funders in the development of new innovations and health technologies, without commensurate investment to identify ‘how’ existing and effective interventions can best implemented to benefit patients and the community [5]. Consequently, there has been a call for more implementation research to enhance the implementation of effective innovations [5].

Current research processes to improve interventions and implementation strategies, however, are often slow, inefficient, and at times wasteful. In part this is because research is not co-ordinated, or informed by prior syntheses of the evidence-base, which can lead to unnecessary duplication or investigation of low priority research questions [6]. Furthermore, research is undertaken in a way that is difficult to synthesise, such as the use of different research designs or outcome measures inhibiting the contribution to studies to the collective advancement of knowledge [7]. Rather than rely on the accumulation of research evidence, more deliberate, and efficient methods to optimisation to the impact of health interventions, are required.

Optimisation is defined [8] as “a deliberate, iterative and data-driven process to improve a health intervention and/or its implementation to meet stakeholder-defined public health impacts within resource constraints”. Such constraints may include time, cost, or intervention complexity [9]. Different aspects of an intervention or its implementation may be optimised to improve the potential impact. This could, for example, include changes to specific components of an intervention or implementation strategy to improve its effectiveness, reduce its costs, or improve its reach. Modifications of the intervention or strategy may relate to the dose, mode of delivery, or component removal, addition or strengthening.

There are a range of models and frameworks that have been proposed to help guide the optimisation process [2, 3]. Among the most common are the Multiphase Optimisation Strategy (MOST) [10], and the Johns Hopkins Quality and Safety Research Group translating evidence into practice model [11]. A recent systematic review of such frameworks suggests optimisation is typically a cyclic process that involves multiple or ongoing evaluations of the intervention and/or implementation strategies, modifications and re-testing these under certain constraint considerations until a pre-specified outcome (e.g. effectiveness, adoption) is achieved [1]. This process occurs across multiple phases of testing intervention components or implementation strategies, examining their effects on outcomes valued by health services, patients, or the community; and identifying opportunities for improvement to enhance their ‘real world’ impact [2]. Optimisation, for example, may involve removing components that do not contribute to the desired effects, adding components that may further enhance effects, or modifying how interventions are delivered to reduce their cost or improve end-user acceptability or experience. Through often repeated processes of experimentation and refinement, more optimal forms of healthcare can be achieved [2].

Despite the existence of broad guidance regarding optimisation processes the specific research designs and methods used to optimise interventions are often unspecified or varied [2, 3]. While most research designs could arguably be used to inform optimisation, certain research designs have been recommended as particularly appropriate in evaluating optimisation. For instance, when optimising the individual components of a health intervention to identify the “best” combination of components within pre-defined constraints, designs allowing direct comparison of the effects of different components are particularly favourable. Factorial and fractional factorial designs have been recommended for optimisation by several frameworks [9, 10, 12] as they allow simultaneous evaluation of multiple intervention components and their interactions while maintaining or reducing the required sample size.

Optimisation, particularly of implementation strategies, may also occur in the context of routine health service delivery as part of quality improvement processes. In these scenarios, different optimisation design selection considerations may be needed compared to those undertaken by academic institutions in laboratories. For example, implementation strategies for routine health service delivery frequently employ clustered designs to reduce contamination, where entire clinics, schools, or hospitals are the unit of randomisation. In these circumstances, the use of some of the recommended trial designs for optimisation, such as factorial designs, may prove particularly challenging due to increased sample and cluster size requirements or the challenges of random assignment of some (e.g. policy level) implementation strategies.

Despite existing guidance regarding efficient trial designs for optimising health interventions, specific advice on the strengths and limitations of these designs, and circumstances in which they are best suited, is limited. Therefore, the aim of this scoping review is to identify what research designs have been used to optimise health and implementation interventions, under what constraints and outcome measures, and whether the designs differ between trials aiming to optimise interventions and implementation strategies. A scoping review methodology was considered the most appropriate review type to address our research question as there has been a paucity of literature on this topic and our primary aim is focused on identifying gaps in the literature.

Review questions

1. What study designs are being used to evaluate the optimisation of health interventions and implementation strategies?
2. What are the constraints used?
3. Do the designs used to optimise interventions and implementation strategies differ?

## Methods

This scoping review was conducted in accordance with the Joanna Briggs Institute (JBI) methodology for scoping reviews [13] and is reported in accordance with the Preferred Reporting Items for Systematic Reviews and Meta-analyses (PRISMA) checklist for scoping reviews (see completed checklist Supplementary file 1) [14].

### Protocol and registration

A protocol was written for this scoping review prior to the conduct of the full search. It can be accessed at doi: 10.17605/OSF.IO/5AMU4.

### Eligibility criteria

Eligible studies were those that were conducted in a health or community setting, aimed to optimise any type of health or implementation intervention, were peer reviewed, and published in English within the last decade (i.e., 2013 to 2023). Any quantitative, experimental design (i.e. testing or evaluating an effect of an intervention or strategy) was considered eligible as long as they stated that optimising a health intervention or implementation strategy was the main goal of the research. For the purposes of this review, optimisation was considered when the main aim of the study was to investigate the effect of the intervention or implementation strategies while limiting or minimising pre-defined constraints, which is in line with definitions of optimisation [8]. Dosing studies, which assess the optimal dose of a drug against side effects, were not included as they have a notably different set of trial properties and purpose to implementation trials and other health intervention trials. Studies were excluded if they were not the primary outcome paper (e.g. excludes cost evaluations, secondary analyses), observational, or qualitative (i.e., not testing effects of an external intervention via an experimental design), focused on non-human participants (e.g. mice), or pilot/feasibility studies.

### Information sources

The following bibliographic databases were searched from 2013 to 2024: Medline, EMBASE, CINAHL, and ProQuest Nursing and Allied Health Source. The International Clinical Trials Registry Platform, and the Australian New Zealand Clinical Trials Registry were also searched to identify relevant trials. All databases were searched on 4th April 2025.

### Search

Search terms were based on titles and abstracts of relevant articles and the search strategies used by similar reviews focusing on optimisation [1] (see Supplementary File 2). A pilot review covering years 2013 and 2014 was run to assess the suitability of the search strategy and scoping review methodology.

### Selection of sources of evidence

Following the search, all identified citations were uploaded into EndNote 20 [15] and duplicates removed. Citations were then imported into the web-based systematic software Covidence [16]. Titles and abstracts were screened independently for eligibility by a pair of reviewers (including: EN, AH, AZ, BP, JD, CL, AS, ER-G, SD, TB). The full text of the potentially eligible sources were retrieved and screened by two independent reviewers (including: EN, AH, JD, TB). Reasons for exclusion were reported, and any disagreement between the two reviewers was resolved either via discussion or a third reviewer. Data were extracted by one reviewer using a study specific, pre-piloted, data extraction tool (including: EN, AH, JD). For 36% of the studies, data were dual extracted independently to reduce errors [17].

### Data items

Data were collected and managed using the REDCap electronic data capture tools hosted at Hunter Medical Research Institute [18, 19]. The data extracted included the title, first author, year of publication, country, aim of the study, whether a framework was used and its name, the study design, whether the study was randomised or non-randomised, number of arms, number of clusters, total sample size (at randomisation), control type (e.g. active, placebo, usual care), statistical framework (e.g. frequentist, Bayesian, both), setting, optimisation target (evidence based intervention and/or implementation strategy) [20], definition of optimisation success, whether optimisation was successful, optimisation constraints, author-reported strengths and impediments of the study design.

### Synthesis of results

A matrix of the experimental designs used in the eligible optimisation trials is presented. The optimisation constraints were mapped against those noted by identified frameworks: time, cost, or complexity [9] or inductively grouped. Considering that the intervention and implementation strategies are quite different, whether the optimisation was occurring to the intervention, implementation strategy, or both, was included to determine what designs were being used to optimise for interventions vs. implementation strategies and whether these differed. The frequency of the used designs, along with the respective optimisation constraints, was tabulated. This provided an evidence map of the most common designs used to optimise health and implementation interventions and under what constraints such designs were used. We discuss gaps in the literature and potential future research.

## Results

**Fig. 1:**
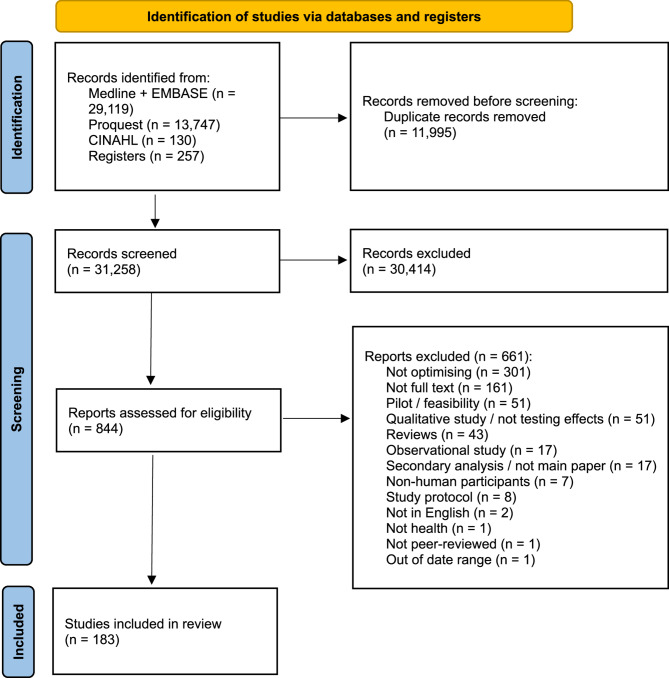
PRISMA flowchart of the scoping review study selection

Forty-three thousand two hundred fifty-three studies were identified, 31,258 screened, and 183 were eligible and included (Fig. 1). The most common reasons for exclusion were ‘Not optimising’ (*n* = 301), ‘Not full text’ (*n* = 161), and ‘Pilot/feasibility’ (*n* = 51).

The characteristics of eligible studies are summarised in Table 1. 77% of studies were optimising interventions, 21% implementation strategies, and 1% both. 24% of studies used an optimisation framework. Less than 20 studies were identified per year prior to 2022, and more than 20 studies were identified per year during and after 2022. Most studies were performed in a community health (50%) or hospital (28%) setting. Only 11% of studies provided a pre-defined criteria to assess the success of optimisation or defined an outcome threshold where optimisation would be considered achieved. The country location of the trial was varied, with no single country representing > 50% of trial locations. The data extracted for the individual sources of evidence are listed in Supplementary File 3.

**Table 1:** Characteristics of the sources of evidence by optimisation target

		Optimisation target *n* (%)		
Characteristic	Overall *N* = 183	Implementation *N* = 39	Intervention *N* = 142	Both *N* = 2
Year of publication				
2013	15 (8.2%)	3 (7.7%)	11 (7.7%)	1 (50%)
2014	14 (7.7%)	1 (2.6%)	12 (8.5%)	1 (50%)
2015	11 (6.0%)	2 (5.1%)	9 (6.3%)	0 (0%)
2016	4 (2.2%)	2 (5.1%)	2 (1.4%)	0 (0%)
2017	6 (3.3%)	2 (5.1%)	4 (2.8%)	0 (0%)
2018	7 (3.8%)	0 (0%)	7 (4.9%)	0 (0%)
2019	17 (9.3%)	5 (13%)	12 (8.5%)	0 (0%)
2020	15 (8.2%)	4 (10%)	11 (7.7%)	0 (0%)
2021	17 (9.3%)	3 (7.7%)	14 (9.9%)	0 (0%)
2022	32 (17%)	9 (23%)	23 (16%)	0 (0%)
2023	22 (12%)	1 (2.6%)	21 (15%)	0 (0%)
2024	23 (13%)	7 (18%)	16 (11%)	0 (0%)
Setting				
Community health	92 (50%)	18 (46%)	72 (51%)	2 (100%)
Hospitals	51 (28%)	21 (54%)	30 (21%)	0 (0%)
General population	20 (11%)	0 (0%)	20 (14%)	0 (0%)
Education	11 (6.0%)	0 (0%)	11 (7.7%)	0 (0%)
Clinical	7 (3.8%)	0 (0%)	7 (4.9%)	0 (0%)
Other	2 (1%)	0 (0%)	2 (1.4%)	0 (0%)
Optimisation framework used	44 (24%)	14 (36%)	30 (21%)	0 (0%)
MOST	28 (15%)	1 (2.6%)	27 (19%)	0 (0%)
PDSA	11 (6.0%)	9 (23%)	2 (1.4%)	0 (0%)
Lean	3 (1.6%)	3 (7.7%)	0 (0%)	0 (0%)
Model for improvement^1^	1 (0.5%)	1 (2.6%)	0 (0%)	0 (0%)
Optimisation success defined	20 (11%)	7 (18%)	13 (9.2%)	0 (0%)
Optimisation successful	134 (73%)	27 (69%)	105 (74%)	2 (100%)
Study design				
Factorial	69 (38%)	9 (23%)	58 (41%)	2 (100%)
Fractional factorial	8 (4.4%)	1 (2.6%)	7 (4.9%)	0 (0%)
RCT^2^	46 (25%)	3 (7.7%)	43 (30%)	0 (0%)
Pre-post	22 (12%)	18 (46%)	4 (2.8%)	0 (0%)
Crossover	16 (8.7%)	2 (5.1%)	14 (9.9%)	0 (0%)
Cluster RCT^2^	7 (3.8%)	3 (7.7%)	4 (2.8%)	0 (0%)
SMART	5 (2.7%)	0 (0%)	5 (3.5%)	0 (0%)
Other	10 (5.5%)	3 (7.7%)	7 (4.9%)	0 (0%)
Adaptive design used	2 (1.1%)	0 (0%)	2 (1.4%)	0 (0%)
Statistical framework				
Frequentist	181 (99%)	39 (100%)	140 (99%)	2 (100%)
Bayesian	2 (1.1%)	0 (0%)	2 (1.4%)	0 (0%)
Country				
USA	69	13	56	0
UK	32	4	28	0
Australia	13	5	6	2
Canada	10	4	6	0
Netherlands	9	1	8	0
Spain	6	1	5	0
China	5	1	4	0
France	5	0	5	0
Germany	5	2	3	0
India	5	1	4	0
Sweden	5	1	4	0
Other^3^	37	8	29	0

^1^One study used both PDSA and Model for improvement

^2^RCT Randomised control trial

^3^Other countries include: Switzerland, Belgium, Ireland, Italy, Romania, Botswana, Brazil, Côte d’Ivoire, Cameroon, Poland, Egypt, Ethiopia, Finland, Israel, Japan, Malawi, New Zealand, Pakistan, Rwanda, Nigeria, Singapore, South Africa, Austria, United Arab Emirates, Taiwan (Republic of China), Puerto Rico

Table 2 provides an overview of the main designs and optimisation constraints used by included studies, summarised by the type of intervention (i.e., health intervention or implementation strategies). The main designs used for optimisation in intervention trials were factorial (41%) and parallel RCTs (30%). The most common designs for implementation trials were pre-post (46%) and factorial (23%). Two (1%) studies used an adaptive design, and two (1%) studies used a Bayesian statistical framework to evaluate optimisation.

**Table 2:** Frequency of optimisation constraints (%) by optimisation target and study design

Optimisation target		Constraint (*n* %)					
Study design	*N* studies	Cost	Time	Complexity	Burden	All active components	Other
Implementation	39	18 (46%)	6 (15%)	8 (21%)	5 (13%)	15 (38%)	2 (5.1%)
Factorial	9	2	0	2	0	8	0
Fractional factorial	1	0	0	0	0	1	0
Cluster RCT^1^	3	2	2	1	0	1	0
RCT^1^	3	3	1	0	1	0	0
Crossover	2	1	0	0	0	1	0
Pre-post	18	9	2	5	2	4	2
Other	3	1	1	0	2	0	0
Intervention	142	60 (42%)	14 (9.9%)	7 (4.9%)	50 (35%)	53 (37%)	2 (1.4%)
Factorial	58	17	2	2	10	38	1
Fractional factorial	7	2	0	0	0	5	1
Cluster RCT^1^	4	2	1	0	1	1	0
RCT^1^	43	30	6	2	21	2	0
Crossover	14	2	1	0	11	2	0
Pre-post	4	2	2	2	4	0	0
SMART	5	2	2	1	2	1	0
Other	7	3	0	0	1	4	0
Both	2	0 (0%)	0 (0%)	0 (0%)	0 (0%)	1 (50%)	0 (0%)
Factorial	2	0	0	0	0	1	0
Total	183	78 (43%)	20 (11%)	15 (8.2%)	55 (30%)	69 (38%)	4 (2.2%)

^1^RCT Randomised control trial

The most common optimisation constraint was cost (financial/resource cost) (43%), followed by all active components (38%), burden (e.g. patient side effects) (30%), time (e.g. treatment completed within a semester) (11%), then complexity (affecting the difficulty in delivering the treatment) (8.2%). The all active components criterion is where the researchers seeks to keep in only effective components, removing low value or ineffective components [9]. Implementation trials had cost as the most common constraint (46%) and burden as the least common constraint (13%), whereas intervention trials had cost as the most common constraint (42%) and complexity as the least common constraint (4.9%).

## Discussion

This scoping review identified 183 studies that aimed to optimise an intervention and/or an implementation strategy. A greater number of studies focused on optimising health interventions (*n* = 142, 77%) than implementation strategies (*n* = 39, 21%). The most common research designs employed overall were factorial and randomised control trials, but this was not consistent across health intervention and implementation strategy studies. Pre-post designs were the most common research design for implementation trials (46%). Trials frequently reported low power and confounding as impediments to evaluating optimisation.

The use of optimisation trials may be increasing over time with less than 20 studies identified per year pre-2022, and more than 20 studies identified per year from 2022 onwards. This suggests that researchers are starting to address the issue with intervention and implementation effect attenuation, by striving to balance the impact of interventions within the real-world constraints they are to be delivered. However, very few trials (24%) were using optimisation frameworks and defining optimisation success a-priori (11%). Most trials communicated success, even though they had not defined success in specific terms or saw inconclusive results for their primary outcome. Instead of providing a definition of optimisation success, such as *“increase the frontline nursing adoption rate of a new PACU-to-Med/Surg handoff process to 100% within 90 days”* by Pino et al. [21], the success authors communicated was often based on significant p-values on any outcome. A general observation made during data extraction was that some trials used a superiority framework but made noninferiority conclusions, judged from high p-values.

The implications of these findings are ambiguous methods and results, making it difficult to use the content for further research, in particular such ambiguity makes it difficult to make meaningful updates to the intervention of implementation strategies that would likely withstand the real-world constraints they will face; thus ultimately contradicting the overall purpose of optimisation. These ambiguous results could be reflective of the low proportion of trials that used optimisation frameworks, which provide specific guidance on how to appropriately evaluate and communicate optimisation.

The results indicate that implementation trials are largely trying to optimise under different constraints to intervention trials. The results suggest that safety and patient burden were primary concerns for interventions whereas cost and complexity was more of a concern for implementation strategies. While burden was not a constraint pre-defined in frameworks, it appeared frequently in the eligible studies. The burden constraint often appeared as side effects to patients, or general intervention/implementation intensity. Unsurprisingly, the consideration of side effects is not common in implementation studies, which, in principle are seeking to implement interventions where these effects are already known. Instead, implementation constraints are more frequently about the ability for the implementation to be scaled to a broad population, continuously, or for a long time. These different constraint considerations may have methodology implications. Methods for evaluating optimisation for implementation strategies may need to consider incorporating economic or cost data, whereas this may not be as important for intervention trials.

Trials optimising the intervention primarily used factorial designs, which is recommended in the MOST framework [9], whereas implementation trials more frequently used pre-post designs, which are not specifically recommended in the MOST framework. Factorial designs enable the direct assessment of the effects of different intervention components, and their interactions and for this reason are recommended as part of optimisation [9]. However, the use of such designs can be complicated, particularly where individual assignment is not possible. It may also not be feasible to administer a variety of different intervention component combinations. Optimisation through repeated, sequential trials, has been seen to be more feasible in such circumstances. Implementation trials were frequently using designs without control groups such as pre-post designs. Such designs are exposed to biases including confounding and are less able to rigorously attribute the effects to a strategy (or its components) on the outcomes it is seeking to change, providing less certainty regarding opportunities for improvement [22]. As implementation trials are complex, often required clustered designs with assignment of units (hospitals, clinics) rather than individuals (patients), the use of randomised controlled trials of factorial trials can represent a particular challenge [7]. Nonetheless, randomised designs are recommended for the evaluation and optimisation of implementation efforts to improve the impact of healthcare, and there are a number of examples where they have been successfully integrated into improvement efforts of health services [6].

The limited use of optimisation frameworks (present only in 24% of studies) was a surprise among a group of studies where optimisation was the goal. This may be due to a lack of awareness in some fields about the specific process of optimization, which seems to be formalised with the first seminal paper on the MOST framework by Collins in 2005 [23], and therefore many may not be familiar with the relevant frameworks and theories of optimisation that exist especially for early studies. Optimisation frameworks, such as MOST framework, can help researchers identify appropriate designs and methods to disentangle treatment effects and avoid confounding. Further, greater use of adaptive designs may help alleviate issues such as a lack of statistical power, often reported in optimisation studies. Adaptive designs are designs that allow trial modifications to occur prior to its conclusion using data gathered during the trial. They offer the potential to increase trial efficiency and power while maintaining acceptable type 1 error [24–26] by offering methods to divert power to better performing treatment components. Confounding may be reduced when using adaptive designs if the increased power and efficiency allow for a control arm to be incorporated. Only two trials identified in the scoping review used adaptive designs and reported the design a facilitator to evaluating optimisation [27, 28].

The use of Bayesian statistical methods may also provide a number of benefits for future optimisation studies. Bayesian framework draw on a combination of prior knowledge and current data to estimate effects [29]. These frameworks offer increased power under certain conditions, for example, when incorporating accurate historical information on treatment component effects [30]. Adaptive designs and Bayesian statistics align with the optimisation process by facilitating the incorporation of learnings and data to guide improvement decisions. In the scoping review, the use of these approaches was low, with 1% using an adaptive design, and 1% using a Bayesian framework to analyse results.

Another option to facilitate optimisation is better co-ordination of research that is being undertaken. Consolidating samples across research groups seeking to optimise a similar intervention or implementation approach, and harmonising key methods to do so can assist with increasing the power by increasing the total number of participants available for analysis as part of a multi-centre trial, or pooled prospective analysis of the findings of multiple independent trials. This method allows for research groups to obtain an adequately sized sample that wouldn’t have been sufficient from any singular area. It can be performed by employing master protocols and ensuring methods and main outcomes are the same to enable data pooling. This method is currently being used in implementation science [31] and has established examples of data analysis procedures [32].

## Limitations

This study had several limitations. The search was limited to single studies. Optimisation efforts that occurred over a series of prospective linked studies may have been missed, unless these were explicitly referred to in the included manuscript (e.g., sequential RCTs across multiple papers). Despite performing an extensive search to systematically identify studies that aimed to optimise interventions or strategies, because optimisation is often not well reported or communicated by authors, we may have missed studies that were optimising. The search was also limited to English studies, as we did not have the resource of expertise to translate non-English studies. This may have reduced the generalisability of our results by introducing language bias [33, 34]. We also acknowledge a deviation from the prospectively published protocol. Specifically, rather than dual data extraction for all included studies dual data extraction occurred for approximately one third of studies. We decided to move to single data extraction given the number of included studies, and the reliability achieved of data extracted in duplicate. Dual data extraction for a subset of studies has been successfully performed by other studies previously [35, 36], but it is acknowledged that the risk of incorrect data extraction increases with this method [37].

## Conclusions

This review summarises design characteristics of trials aiming to optimise health interventions or implementation strategies and what facilitators and impediments to evaluating optimisation these trials encountered. There is a need for the use of frameworks to guide trial design and for a clear definition of optimisation success. It is recommended to consider using alternate methods that may overcome common impediments and align better with optimisation, such as adaptive designs and Bayesian statistics. There exists an opportunity to develop further guidance on how trials can efficiently evaluate optimisation.

## Supplementary Information

Supplementary Material 1.

Supplementary Material 2.

Supplementary Material 3.

## Data Availability

The datasets used and/or analysed during the current study are available from the corresponding author on reasonable request.
