# Supplementary material for: Experimental designs used for optimising the effects of health interventions and implementation strategies: a scoping review
Source: BMC Health Serv Res. 2025 Aug 25;25:1129. doi: 10.1186/s12913-025-13184-9 (PMC12379312; doi:10.1186/s12913-025-13184-9)
Supplement: Supplementary file 2 — Supplementary Material 2. [file 12913_2025_13184_MOESM2_ESM.docx]

**Supplementary File 2:** Search terms

Table S2: Search terms

| Improvement terms (Optimisation, quality improvement, continuous quality improvement) | 1 | optimi* |
| --- | --- | --- |
|  | 2 | continuous quality improvement |
|  | 3 | quality improvement*  or improve* quality  or quality management*  or improve* patient care  or process improvement |
| Context | 4 | human*  or adult*  or adolescent*  or child*  or parent*  or patient*  or male*  or female*  or men  or women  or participant*  or student*  or employee*  or survivor* |
| Designs | 5 | factorial or sequential  or micro-randomi$  or micro randomi$  or system identification  or adaptive  or multi-phase optimi$ strategy  or multiphase optimi$ strategy  or RCT  or c$RCT  or step wedge  or stepped wedge  or quasi-experimental  or quasi experimental  or pragmatic  or latin square  or crossover |
| Rewording for factorial | 6 | (“2 by 2” or “2 x 2” or “2 by 3” or “2 x 3” or “3 by 2” or “3 x 2” or “3 by 3” or “3 x 3”) |
|  | 7 | (1 or 2 or 3) and 4 and (5 or 6) |

(1 or 2 or 3) and (4) and (5 or 6)

Ovid (Medline, embase)

1. (optimi* or quality improvement* or improve* quality or quality management* or improve* patient care or process improvement).ti,ab.
2. (human* or adult* or adolescent* or child* or parent* or patient* or male* or female* or men or women or participant* or student* or employee* or survivor*).ti,ab.
3. (factorial or sequential or micro-randomi$ or micro randomi$ or system identification or adaptive or multi-phase optimi$ strategy or multiphase optimi$ strategy or RCT or c*RCT or step wedge or stepped wedge or quasi-experimental or quasi experimental or pragmatic or latin square or crossover).ti,ab.
4. ("2 by 2" or "2 x 2" or "2 by 3" or "2 x 3" or "3 by 2" or "3 x 2" or "3 by 3" or "3 x 2").ti,ab.
5. 1 and 2 and (3 or 4)

Cochrane library (ICTRP, CINAHL, ANZCTR)

“#1 - (optimi* or quality improvement* or improve* quality or quality management* or improve* patient care or process improvement) and (human* or adult* or adolescent* or child* or parent* or patient* or male* or female* or men or women or participant* or student* or employee* or survivor*) and ((factorial or sequential or micro-randomi$ or micro randomi$ or system identification or adaptive or multi-phase optimi$ strategy or multiphase optimi$ strategy or RCT or c*RCT or step wedge or stepped wedge or quasi-experimental or quasi experimental or pragmatic or latin square or crossover) or ("2 by 2" or "2 x 2" or "2 by 3" or "2 x 3" or "3 by 2" or "3 x 2" or "3 by 3" or "3 x 2")):ti,ab,kw”

Proquest (ProQuest Nursing and Allied Health Source)

(optimi* or quality improvement* or improve* quality or quality management* or improve* patient care or process improvement) in “Anywhere except full text – NOFT”

AND

(human* or adult* or adolescent* or child* or parent* or patient* or male* or female* or men or women or participant* or student* or employee* or survivor*) in “Anywhere except full text – NOFT”

AND

((factorial or sequential or micro-randomi$ or micro randomi$ or system identification or adaptive or multi-phase optimi$ strategy or multiphase optimi$ strategy or RCT or c*RCT or step wedge or stepped wedge or quasi-experimental or quasi experimental or pragmatic or latin square or crossover) or ("2 by 2" or "2 x 2" or "2 by 3" or "2 x 3" or "3 by 2" or "3 x 2" or "3 by 3" or "3 x 2")) in “Anywhere except full text – NOFT”
