# Supplementary material for: Experimental designs used for optimising the effects of health interventions and implementation strategies: a scoping review
Source: BMC Health Serv Res. 2025 Aug 25;25:1129. doi: 10.1186/s12913-025-13184-9 (PMC12379312; doi:10.1186/s12913-025-13184-9)
Supplement: Supplementary file 3 — Supplementary Material 3. [file 12913_2025_13184_MOESM3_ESM.docx]

**Supplementary File 3:** Characteristics of individual included studies

Table S3: Characteristics of individual included studies

|  |  |  |  |  |  |  | Constraint | | | | |
| --- | --- | --- | --- | --- | --- | --- | --- | --- | --- | --- | --- |
| Reference | First Author | Year of Publication | Study Design | Optimisation target | Optimisation success defined | Optimisation framework | Cost | Time | Complexity | Burden | All active components |
| (1) | Adam Cheng | 2015 | Factorial | Implementation | No |  | No | No | Yes | No | Yes |
| (2) | Adekunle Dawodu | 2013 | Randomised control trial | Intervention | No |  | No | No | No | Yes | No |
| (3) | Ahmed Taher | 2020 | Pre-post | Implementation | No | PDSA | Yes | No | Yes | No | No |
| (4) | Aisha K Yousafzai | 2014 | Factorial | Intervention | No |  | No | No | No | No | Yes |
| (5) | Alan R. Tait | 2013 | Fractional factorial | Intervention | No |  | No | No | No | No | Yes |
| (6) | Alexander Blood | 2024 | Randomised control trial | Intervention | No |  | No | No | No | No | No |
| (7) | Alexandra Wright‑Hughes | 2022 | Fractional factorial | Implementation | No | MOST | No | No | No | No | Yes |
| (8) | Alline Beleigoli | 2020 | Randomised control trial | Intervention | No |  | Yes | No | No | No | Yes |
| (9) | Amanda C. Thompkins | 2014 | Pre-post | Intervention | No |  | No | Yes | Yes | Yes | No |
| (10) | Amanda E. Tanner | 2021 | Factorial | Intervention | No | MOST | No | No | No | No | Yes |
| (11) | Amanda L Graham | 2020 | Factorial | Intervention | No | MOST | No | No | No | No | Yes |
| (12) | Amar Rangan | 2020 | Randomised control trial | Intervention | No |  | Yes | No | No | Yes | No |
| (13) | Amish Khan | 2023 | Factorial | Intervention | No |  | No | No | No | No | Yes |
| (14) | Amit Kumar | 2022 | Pre-post | Implementation | No | PDSA | No | No | Yes | No | Yes |
| (15) | Amit Pahwa | 2024 | Pre-post | Implementation | No |  | No | No | No | No | Yes |
| (16) | Amy Abernethy | 2013 | Factorial | Both | No |  | No | No | No | No | Yes |
| (17) | Andrew Marshall | 2023 | Factorial | Intervention | No |  | Yes | No | No | No | No |
| (18) | Ann Angiolillo | 2021 | Factorial | Intervention | No |  | No | No | No | Yes | No |
| (19) | Anna Sallis | 2016 | Randomised control trial | Intervention | No |  | Yes | No | No | No | No |
| (20) | Ashley Blom | 2022 | Randomised control trial | Intervention | No |  | Yes | Yes | No | Yes | No |
| (21) | Bonnie Spring | 2020 | Factorial | Intervention | No | MOST | Yes | No | No | No | No |
| (22) | Bruce Guthrie | 2016 | Randomised control trial | Implementation | No |  | Yes | No | No | No | No |
| (23) | C. Jones | 2015 | Factorial | Intervention | No |  | No | No | No | No | Yes |
| (24) | Cagla Kantarcigil | 2020 | Crossover | Intervention | No |  | Yes | No | No | Yes | No |
| (25) | Caitlin Horsham | 2021 | Other | Intervention | No |  | Yes | No | No | No | Yes |
| (26) | Carina Lis ø y | 2024 | Factorial | Intervention | No | MOST | Yes | No | Yes | No | No |
| (27) | Carla Maria Dohrendorf | 2021 | Crossover | Implementation | No |  | No | No | No | No | Yes |
| (28) | Carmina G Valle | 2018 | Factorial | Intervention | No | MOST | No | No | No | No | Yes |
| (29) | Catherine Kimber | 2023 | Factorial | Intervention | No | MOST | Yes | No | No | No | Yes |
| (30) | Chad D. Rethorst | 2024 | Factorial | Intervention | Yes | MOST | No | No | No | No | Yes |
| (31) | Charles Cleland | 2023 | Fractional factorial | Intervention | No | MOST | Yes | No | No | No | No |
| (32) | Charlotte L Edwardson | 2022 | Cluster randomised control trial | Intervention | No |  | Yes | No | No | No | No |
| (33) | Christine Andrews | 2018 | Pre-post | Intervention | No | PDSA | Yes | Yes | No | Yes | No |
| (34) | Christopher B Forrest | 2013 | Factorial | Implementation | No |  | No | No | No | No | Yes |
| (35) | Christopher E. Cox | 2024 | Factorial | Intervention | Yes | MOST | No | No | Yes | Yes | No |
| (36) | Christopher M. Celano | 2018 | Factorial | Intervention | No | MOST | No | No | No | No | Yes |
| (37) | Christopher Sundström | 2022 | Factorial | Intervention | No | MOST | No | No | No | No | Yes |
| (38) | Dario Baretta | 2023 | Factorial | Intervention | Yes | MOST | No | No | No | No | No |
| (39) | David Cameron | 2017 | Factorial | Intervention | No |  | No | No | No | Yes | No |
| (40) | David Erlinge | 2013 | Crossover | Intervention | No |  | No | No | No | Yes | No |
| (41) | David L. Wyrick | 2022 | Factorial | Intervention | No | MOST | No | No | No | No | Yes |
| (42) | Deborah L. Zimmerman | 2014 | Crossover | Intervention | No |  | No | No | No | Yes | No |
| (43) | Debra Parker Oliver | 2023 | Crossover | Intervention | No |  | No | No | No | No | Yes |
| (44) | Denise A. O'Connor | 2022 | Factorial | Implementation | No |  | No | No | No | No | Yes |
| (45) | Diana Rodríguez-Espinosa | 2024 | Crossover | Intervention | No |  | No | No | No | Yes | No |
| (46) | Dipen P. Vyas | 2022 | Pre-post | Implementation | Yes | PDSA | No | No | No | No | No |
| (47) | Dominik Glinz | 2015 | Factorial | Intervention | No |  | No | No | No | No | Yes |
| (48) | Edward Roddy | 2021 | Factorial | Intervention | No |  | No | No | No | No | Yes |
| (49) | Edward Watkins | 2023 | Fractional factorial | Intervention | No | MOST | No | No | No | No | Yes |
| (50) | Elvin H. Geng | 2023 | SMART | Intervention | No |  | No | Yes | No | No | No |
| (51) | Felicity A. Pino | 2019 | Pre-post | Implementation | Yes | Lean | No | Yes | No | No | No |
| (52) | Fred Semitala | 2024 | Randomised control trial | Intervention | Yes |  | Yes | No | No | Yes | No |
| (53) | Giuseppe Patti | 2013 | Factorial | Intervention | No |  | No | No | No | Yes | No |
| (54) | Hannes Weise | 2022 | Randomised control trial | Intervention | No |  | Yes | No | No | Yes | No |
| (55) | Hassan Seddik | 2024 | Randomised control trial | Intervention | Yes |  | Yes | No | No | No | No |
| (56) | Ildiko Tombor | 2018 | Factorial | Intervention | No | MOST | No | No | No | No | Yes |
| (57) | Ilse Graat | 2023 | Crossover | Intervention | No |  | No | No | No | Yes | No |
| (58) | J. A. Forrester | 2021 | Pre-post | Implementation | No |  | Yes | No | No | No | No |
| (59) | J. Stevens | 2019 | Pre-post | Implementation | No |  | Yes | No | No | No | No |
| (60) | Jack Dekker | 2013 | Randomised control trial | Intervention | No |  | Yes | No | No | Yes | No |
| (61) | Jaspreet Kaur | 2018 | Factorial | Intervention | No |  | No | No | No | No | Yes |
| (62) | Jaspreet Kaur | 2017 | Factorial | Intervention | No |  | No | No | No | Yes | No |
| (63) | Jean Joel R Bigna | 2014 | Factorial | Intervention | No |  | Yes | Yes | No | No | No |
| (64) | Jeffrey M Pernica | 2022 | Factorial | Intervention | No |  | Yes | No | No | No | No |
| (65) | Jennifer B McClure | 2013 | Factorial | Intervention | No | MOST | No | No | No | No | Yes |
| (66) | Jens Abraham | 2019 | Cluster randomised control trial | Implementation | No |  | No | Yes | Yes | No | Yes |
| (67) | Jeremy D. Krebs | 2018 | Crossover | Intervention | No |  | No | No | No | No | Yes |
| (68) | John C. Fortney | 2015 | Randomised control trial | Intervention | No |  | No | No | No | Yes | No |
| (69) | John C. Fortney | 2021 | SMART | Intervention | No |  | Yes | No | No | Yes | No |
| (70) | John C. Fortney | 2013 | Randomised control trial | Intervention | No |  | Yes | Yes | No | No | No |
| (71) | Jon C. Tilburt | 2022 | Factorial | Intervention | No |  | No | No | No | No | Yes |
| (72) | Jon D Emery | 2017 | Factorial | Implementation | No |  | No | No | No | No | Yes |
| (73) | Jonathan Bisson | 2022 | Randomised control trial | Intervention | Yes |  | Yes | Yes | No | Yes | No |
| (74) | Joy M. Schmitz | 2024 | SMART | Intervention | Yes |  | Yes | No | No | Yes | No |
| (75) | Judy Pa | 2014 | Factorial | Intervention | No |  | No | No | No | No | Yes |
| (76) | Julie Fritz | 2021 | Factorial | Intervention | No | MOST | No | No | No | No | Yes |
| (77) | Juliet M. Foster | 2014 | Factorial | Both | No |  | No | No | No | No | No |
| (78) | Julio Rosenstock | 2013 | Crossover | Intervention | No |  | No | No | No | Yes | No |
| (79) | Justin C. Brown | 2023 | Factorial | Intervention | No |  | No | No | No | No | Yes |
| (80) | Kang-Cheng Su | 2017 | Pre-post | Implementation | No |  | Yes | No | Yes | No | No |
| (81) | Karim Fizazi | 2022 | Factorial | Intervention | No |  | No | No | No | No | Yes |
| (82) | Karina F. M. Tao | 2021 | Crossover | Intervention | No |  | No | No | No | Yes | No |
| (83) | Kathryn Manning | 2019 | Other | Intervention | No |  | Yes | No | No | No | No |
| (84) | Kelly L. Rulison | 2022 | Fractional factorial | Intervention | No | MOST | No | No | No | No | Yes |
| (85) | Kevin L. Schwartz | 2024 | Factorial | Implementation | No |  | No | No | No | No | Yes |
| (86) | Kimber P Richter | 2015 | Randomised control trial | Intervention | No |  | Yes | No | No | No | No |
| (87) | L.L. Kemmeren | 2023 | Randomised control trial | Intervention | No |  | No | No | No | Yes | No |
| (88) | Lauren Bell | 2023 | Micro-randomised | Intervention | No | MOST | No | No | No | No | Yes |
| (89) | Lauren Eberly | 2024 | Other | Intervention | No |  | Yes | No | No | No | No |
| (90) | Lawrence Fisher | 2013 | Randomised control trial | Intervention | No |  | No | No | No | No | Yes |
| (91) | Leanne Morrison | 2014 | Fractional factorial | Intervention | No |  | No | No | No | No | Yes |
| (92) | Lena Rindner | 2023 | Factorial | Intervention | No |  | No | No | No | No | Yes |
| (93) | Liliane Windsor | 2024 | Factorial | Intervention | No |  | Yes | No | No | No | No |
| (94) | Lori Bilello | 2019 | Other | Implementation | No |  | Yes | Yes | No | No | No |
| (95) | Lucinda Bell | 2023 | Factorial | Intervention | No | MOST | No | No | No | No | Yes |
| (96) | Lynne I. Wagner | 2021 | Factorial | Intervention | No | MOST | Yes | No | No | No | No |
| (97) | Marconi Abreu | 2019 | Randomised control trial | Intervention | No |  | No | No | Yes | Yes | No |
| (98) | Marieke de Gier | 2023 | Randomised control trial | Intervention | No |  | Yes | Yes | No | No | No |
| (99) | Mark Neuman | 2024 | Factorial | Implementation | No |  | Yes | No | Yes | No | No |
| (100) | Masatsugu Sakata | 2022 | Factorial | Intervention | No |  | No | No | No | No | Yes |
| (101) | Matilda Berg | 2020 | Factorial | Intervention | No |  | Yes | No | No | Yes | Yes |
| (102) | Matisyahu Shulman | 2024 | Other | Intervention | Yes |  | No | No | No | Yes | No |
| (103) | Melissa B. Gilkey | 2022 | Cluster randomised control trial | Implementation | No |  | Yes | Yes | No | No | No |
| (104) | Michele Heisler | 2021 | Cluster randomised control trial | Intervention | No |  | No | No | No | Yes | No |
| (105) | Mohamed Abdel‑Fattah | 2022 | Randomised control trial | Intervention | Yes |  | No | No | No | Yes | No |
| (106) | Mohamed E. Abdel-Ghaffar | 2022 | Randomised control trial | Intervention | Yes |  | No | No | No | Yes | No |
| (107) | Nadine Köhle | 2021 | Randomised control trial | Intervention | No |  | Yes | No | No | No | No |
| (108) | Nancy E. Sherwood | 2022 | SMART | Intervention | No |  | No | No | No | No | Yes |
| (109) | Nancy Rumbaugh Whitesell | 2019 | Fractional factorial | Intervention | No | MOST | Yes | No | No | No | No |
| (110) | Natasha Jennings | 2015 | Randomised control trial | Implementation | No |  | Yes | No | No | No | No |
| (111) | Nikesh Thiruchelvam | 2024 | Crossover | Intervention | No |  | No | No | No | Yes | No |
| (112) | Noah M Ivers | 2020 | Randomised control trial | Intervention | No |  | Yes | No | No | No | No |
| (113) | O. Manzi | 2014 | Pre-post | Implementation | No |  | Yes | No | No | No | No |
| (114) | Okon Essien | 2017 | Randomised control trial | Intervention | No |  | Yes | No | Yes | No | No |
| (115) | Oliver Thomas Bur | 2022 | Factorial | Intervention | No |  | No | No | No | No | Yes |
| (116) | Olivier Mimoz | 2015 | Factorial | Intervention | No |  | No | No | No | No | Yes |
| (117) | Padmanabhan Ramnarayan | 2022 | Randomised control trial | Intervention | No |  | No | No | No | Yes | No |
| (118) | Pascal M. Lavoie | 2015 | Factorial | Intervention | No |  | No | No | No | No | Yes |
| (119) | Patricia Martínez-Ibáñez | 2022 | Randomised control trial | Intervention | No |  | Yes | No | No | No | No |
| (120) | Patryk Łakuta | 2022 | Factorial | Intervention | No |  | No | No | No | No | No |
| (121) | Peter Anderson | 2016 | Factorial | Implementation | No |  | No | No | No | No | Yes |
| (122) | Peter Pirolli | 2017 | Fractional factorial | Intervention | No |  | No | No | No | No | Yes |
| (123) | Peter Szilagyi | 2020 | Randomised control trial | Intervention | No |  | Yes | No | No | No | No |
| (124) | Petra Denig | 2014 | Factorial | Intervention | No |  | No | No | No | No | Yes |
| (125) | Pinki Meena | 2024 | Pre-post | Intervention | Yes | PDSA | Yes | No | No | Yes | No |
| (126) | Predrag Klasnja | 2019 | Micro-randomised | Intervention | No |  | No | No | No | No | Yes |
| (127) | Rebecca Palmer | 2019 | Randomised control trial | Intervention | No |  | Yes | No | No | No | No |
| (128) | Richard Holland | 2014 | Randomised control trial | Intervention | No |  | Yes | No | No | Yes | No |
| (129) | Robert J. Fortuna | 2014 | Randomised control trial | Intervention | No |  | Yes | No | No | No | No |
| (130) | Roberto Collado-Borrell | 2020 | Pre-post | Intervention | No |  | No | No | Yes | Yes | No |
| (131) | Roger D James | 2013 | Factorial | Intervention | No |  | No | No | No | Yes | No |
| (132) | Ronald J. Prinz | 2022 | Randomised control trial | Intervention | Yes |  | Yes | No | No | Yes | No |
| (133) | Sabita Uthaya | 2016 | Factorial | Intervention | No |  | Yes | No | No | No | Yes |
| (134) | Samarth Shukla | 2020 | Pre-post | Implementation | Yes | PDSA | Yes | Yes | No | No | No |
| (135) | Sandra C. Hayes | 2013 | Randomised control trial | Intervention | No |  | No | No | No | Yes | No |
| (136) | Sanjoti Parekh | 2014 | Factorial | Intervention | No |  | No | Yes | No | Yes | No |
| (137) | Sara Kalkhoran | 2019 | Randomised control trial | Intervention | No |  | Yes | No | No | No | No |
| (138) | Sarah L. Cutrona | 2018 | Factorial | Intervention | No |  | Yes | No | No | No | No |
| (139) | Sean M. Cullen | 2022 | Pre-post | Implementation | Yes | PDSA | No | No | Yes | No | Yes |
| (140) | Seth Himelhoch | 2024 | Factorial | Intervention | No |  | No | No | No | No | Yes |
| (141) | Sharon Landesman Ramey | 2021 | Factorial | Intervention | No |  | Yes | No | No | Yes | No |
| (142) | Shinnona AlHarthy | 2024 | Pre-post | Implementation | No |  | No | No | Yes | No | No |
| (143) | Shinnona AlHarthy | 2024 | Pre-post | Implementation | No | Lean | Yes | No | No | Yes | No |
| (144) | Shivan J. Mehta | 2019 | Randomised control trial | Intervention | No |  | Yes | No | No | No | No |
| (145) | Shu-Yu Bi | 2022 | Pre-post | Implementation | Yes | PDSA | No | No | No | No | Yes |
| (146) | Simon Gilbody | 2015 | Randomised control trial | Intervention | No |  | Yes | No | No | No | No |
| (147) | Simon J. Stanworth | 2022 | Factorial | Implementation | No |  | Yes | No | No | No | Yes |
| (148) | Simone Peart Boyce | 2019 | Randomised control trial | Implementation | No |  | Yes | Yes | No | Yes | No |
| (149) | Simonetta Scalvini | 2013 | Other | Implementation | No |  | No | No | No | Yes | No |
| (150) | Siobhan M. Phillips | 2022 | Factorial | Intervention | No | MOST | Yes | No | No | No | No |
| (151) | Solomon Tesfaye | 2022 | Crossover | Intervention | No |  | No | No | No | Yes | No |
| (152) | Sophie M.C. Green | 2023 | Factorial | Intervention | Yes | MOST | No | No | No | No | Yes |
| (153) | Stephanie G. Kerrigan | 2021 | Factorial | Intervention | No |  | Yes | No | No | No | Yes |
| (154) | Steven Bernstein | 2023 | Factorial | Intervention | No | MOST | No | No | No | No | Yes |
| (155) | Steven S. Fu | 2023 | SMART | Intervention | No |  | No | Yes | Yes | No | No |
| (156) | Stuart A. Little | 2014 | Factorial | Intervention | No |  | No | No | No | No | Yes |
| (157) | Sullafa Kadura | 2024 | Pre-post | Implementation | No |  | No | No | No | Yes | No |
| (158) | Sunil Kripalani | 2019 | Randomised control trial | Intervention | No |  | Yes | No | No | No | No |
| (159) | Tatsuo Akechi | 2024 | Factorial | Intervention | No | MOST | Yes | No | No | No | Yes |
| (160) | Tero Kortekangas | 2019 | Randomised control trial | Intervention | No |  | No | Yes | No | Yes | No |
| (161) | Tianshu Zeng | 2021 | Randomised control trial | Intervention | No |  | Yes | No | No | Yes | No |
| (162) | Tim Colbourn | 2013 | Factorial | Implementation | No |  | No | No | No | No | Yes |
| (163) | Timothy I. Morgenthaler | 2014 | Randomised control trial | Intervention | No |  | Yes | No | No | No | No |
| (164) | Trung Du | 2022 | Pre-post | Implementation | Yes | PDSA | Yes | No | No | No | No |
| (165) | Tsai-Wing Ow | 2024 | Crossover | Implementation | No |  | Yes | No | No | No | No |
| (166) | Usha Chakravarthy | 2015 | Factorial | Intervention | No |  | Yes | No | No | Yes | No |
| (167) | Valy Fontil | 2023 | Cluster randomised control trial | Implementation | No |  | Yes | No | No | No | No |
| (168) | Varsha Ramineni | 2023 | Randomised control trial | Intervention | No |  | No | Yes | No | No | No |
| (169) | Vera Yakovchenko | 2020 | Other | Implementation | No | Lean | No | No | No | Yes | No |
| (170) | Veronique Orcel | 2024 | Cluster randomised control trial | Intervention | No |  | Yes | No | No | No | No |
| (171) | Vicky Stergiopoulos | 2015 | Randomised control trial | Intervention | No |  | Yes | No | No | No | No |
| (172) | Vincent A van Vugt | 2019 | Randomised control trial | Intervention | No |  | Yes | No | No | Yes | No |
| (173) | Virginie Dauphinot | 2020 | Crossover | Intervention | No |  | No | Yes | No | Yes | No |
| (174) | Wee Meng Han | 2021 | Pre-post | Implementation | No | PDSA | Yes | No | No | No | No |
| (175) | Weiwei Beckerleg | 2020 | Pre-post | Implementation | Yes | PDSA & Model for Improvement | No | No | No | No | No |
| (176) | Yajie Wang | 2023 | Randomised control trial | Intervention | No |  | No | No | No | Yes | No |
| (177) | Yelena P. Wu | 2019 | Cluster randomised control trial | Intervention | No | MOST | No | Yes | No | No | Yes |
| (178) | Yeqing Gu | 2020 | Factorial | Intervention | No |  | No | No | No | No | Yes |
| (179) | Ying Jiang | 2022 | Randomised control trial | Intervention | No |  | Yes | No | No | Yes | No |
| (180) | Yolanda Ramallo-Fariña | 2021 | Factorial | Intervention | No |  | Yes | No | No | No | Yes |
| (181) | Yuan Wan | 2022 | Crossover | Intervention | No |  | No | No | No | Yes | No |
| (182) | Zachary I. Whinnett | 2019 | Crossover | Intervention | No |  | Yes | No | No | No | No |
| (183) | Zahra Rooddehghan | 2023 | Other | Intervention | No |  | No | No | No | No | Yes |

**References**

1. Cheng A, Brown LL, Duff JP, Davidson J, Overly F, Tofil NM, et al. Improving cardiopulmonary resuscitation with a CPR feedback device and refresher simulations (CPR CARES Study): a randomized clinical trial. JAMA Pediatr. 2015 Feb;169(2):137–44.

2. Dawodu A, Saadi HF, Bekdache G, Javed Y, Altaye M, Hollis BW. Randomized controlled trial (RCT) of vitamin D supplementation in pregnancy in a population with endemic vitamin D deficiency. J Clin Endocrinol Metab. 2013 Jun;98(6):2337–46.

3. Taher A, Magcalas FW, Woolner V, Casey S, Davies D, Chartier LB. Quality improvement initiative for improved patient communication in an ED rapid assessment zone. Emerg Med J. 2020 Dec;37(12):811–8.

4. Yousafzai AK, Rasheed MA, Rizvi A, Armstrong R, Bhutta ZA. Effect of integrated responsive stimulation and nutrition interventions in the Lady Health Worker programme in Pakistan on child development, growth, and health outcomes: a cluster-randomised factorial effectiveness trial. Lancet. 2014 Oct 4;384(9950):1282–93.

5. Tait AR, Voepel-Lewis T, Nair VN, Narisetty NN, Fagerlin A. Informing the Uninformed: Optimizing the Consent Message Using a Fractional Factorial Design. JAMA Pediatrics. 2013 Jul 1;167(7):640–6.

6. Blood AJ, Chang LS, Hassan S, Chasse J, Stern G, Gabovitch D, et al. Randomized Evaluation of a Remote Management Program to Improve Guideline-Directed Medical Therapy: The DRIVE Trial. Circulation. 2024 Jun 4;149(23):1802–11.

7. Wright-Hughes A, Willis TA, Wilson S, Weller A, Lorencatto F, Althaf M, et al. A randomised fractional factorial screening experiment to predict effective features of audit and feedback. Implementation Science. 2022 May 26;17(1):34.

8. Beleigoli A, Andrade AQ, Diniz MDF, Ribeiro AL. Personalized Web-Based Weight Loss Behavior Change Program With and Without Dietitian Online Coaching for Adults With Overweight and Obesity: Randomized Controlled Trial. J Med Internet Res. 2020 Nov 5;22(11):e17494.

9. Thompkins AC, Chauveron LM, Harel O, Perkins DF. Optimizing violence prevention programs: an examination of program effectiveness among urban high school students. J Sch Health. 2014 Jul;84(7):435–43.

10. Tanner AE, Guastaferro KM, Rulison KL, Wyrick DL, Milroy JJ, Bhandari S, et al. A Hybrid Evaluation-Optimization Trial to Evaluate an Intervention Targeting the Intersection of Alcohol and Sex in College Students and Simultaneously Test an Additional Component Aimed at Preventing Sexual Violence. Ann Behav Med. 2021 Nov 18;55(12):1184–7.

11. Graham AL, Papandonatos GD, Jacobs MA, Amato MS, Cha S, Cohn AM, et al. Optimizing Text Messages to Promote Engagement With Internet Smoking Cessation Treatment: Results From a Factorial Screening Experiment. J Med Internet Res. 2020 Apr 2;22(4):e17734.

12. Rangan A, Brealey SD, Keding A, Corbacho B, Northgraves M, Kottam L, et al. Management of adults with primary frozen shoulder in secondary care (UK FROST): a multicentre, pragmatic, three-arm, superiority randomised clinical trial. The Lancet. 2020 Oct 3;396(10256):977–89.

13. Khan AM, Piccirillo J, Kallogjeri D, Piccirillo JF. Efficacy of Combined Visual-Olfactory Training With Patient-Preferred Scents as Treatment for Patients With COVID-19 Resultant Olfactory Loss: A Randomized Clinical Trial. JAMA Otolaryngol Head Neck Surg. 2023 Feb 1;149(2):141–9.

14. Kumar A, Kumar R, Gupta AK, Kishore S, Kumar M, Ahmar R, et al. Improvement of Hand Hygiene Compliance Using the Plan-Do-Study-Act Method: Quality Improvement Project From a Tertiary Care Institute in Bihar, India. Cureus. 2022 Jun;14(6):e25590.

15. Pahwa A, Rajendran A, Castellanos S, Wu L, Prichett L, Huang S, et al. OslerCare: Modifying timing and delivery of feedback to impact resident lab ordering practices. J Hosp Med. 2024 Aug;19(8):755–60.

16. Abernethy AP, Currow DC, Shelby-James T, Rowett D, May F, Samsa GP, et al. Delivery strategies to optimize resource utilization and performance status for patients with advanced life-limiting illness: results from the “palliative care trial” [ISRCTN 81117481]. J Pain Symptom Manage. 2013 Mar;45(3):488–505.

17. Marshall ASJ, Scrivens A, Bell JL, Linsell L, Hardy P, Yong J, et al. Assessment of infant position and timing of stylet removal to improve lumbar puncture success in neonates (NeoCLEAR): an open-label, 2 × 2 factorial, randomised, controlled trial. The Lancet Child & Adolescent Health. 2023 Feb 1;7(2):91–100.

18. Angiolillo AL, Schore RJ, Kairalla JA, Devidas M, Rabin KR, Zweidler-McKay P, et al. Excellent Outcomes With Reduced Frequency of Vincristine and Dexamethasone Pulses in Standard-Risk B-Lymphoblastic Leukemia: Results From Children’s Oncology Group AALL0932. J Clin Oncol. 2021 May 1;39(13):1437–47.

19. Sallis A, Bunten A, Bonus A, James A, Chadborn T, Berry D. The effectiveness of an enhanced invitation letter on uptake of National Health Service Health Checks in primary care: a pragmatic quasi-randomised controlled trial. BMC Fam Pract. 2016 Mar 24;17:35.

20. Blom AW, Lenguerrand E, Strange S, Noble SM, Beswick AD, Burston A, et al. Clinical and cost effectiveness of single stage compared with two stage revision for hip prosthetic joint infection (INFORM): pragmatic, parallel group, open label, randomised controlled trial. BMJ. 2022 Oct 31;379:e071281.

21. Spring B, Pfammatter AF, Marchese SH, Stump T, Pellegrini C, McFadden HG, et al. A Factorial Experiment to Optimize Remotely Delivered Behavioral Treatment for Obesity: Results of the Opt-IN Study. Obesity (Silver Spring). 2020 Sep;28(9):1652–62.

22. Guthrie B, Kavanagh K, Robertson C, Barnett K, Treweek S, Petrie D, et al. Data feedback and behavioural change intervention to improve primary care prescribing safety (EFIPPS): multicentre, three arm, cluster randomised controlled trial. BMJ. 2016 Aug 18;354:i4079.

23. Jones C, Eddleston J, McCairn A, Dowling S, McWilliams D, Coughlan E, et al. Improving rehabilitation after critical illness through outpatient physiotherapy classes and essential amino acid supplement: A randomized controlled trial. J Crit Care. 2015 Oct;30(5):901–7.

24. Kantarcigil Cagla, Kim Min Ku, Chang Taehoo, Craig Bruce A., Smith Anne, Lee Chi Hwan, et al. Validation of a Novel Wearable Electromyography Patch for Monitoring Submental Muscle Activity During Swallowing: A Randomized Crossover Trial. Journal of Speech, Language, and Hearing Research. 2020 Oct 16;63(10):3293–310.

25. Horsham C, Baade P, Kou K, O’Hara M, Sinclair C, Loescher LJ, et al. Optimizing Texting Interventions for Melanoma Prevention and Early Detection: A Latin Square Crossover RCT. Am J Prev Med. 2021 Sep;61(3):348–56.

26. Lisøy C, Neumer SP, Adolfsen F, Ingul JM, Potulski Rasmussen LM, Wentzel-Larsen T, et al. Optimizing indicated cognitive behavioral therapy to prevent child anxiety and depression: A cluster-randomized factorial trial. Behav Res Ther. 2024 May;176:104520.

27. Dohrendorf CM, Unkel S, Scheithauer S, Kaase M, Meier V, Fenz D, et al. Reduced Clostridioides difficile infections in hospitalised older people through multiple quality improvement strategies. Age Ageing. 2021 Nov 10;50(6):2123–32.

28. Valle CG, Queen TL, Martin BA, Ribisl KM, Mayer DK, Tate DF. Optimizing Tailored Communications for Health Risk Assessment: A Randomized Factorial Experiment of the Effects of Expectancy Priming, Autonomy Support, and Exemplification. J Med Internet Res. 2018 Mar 1;20(3):e63.

29. Kimber C, Sideropoulos V, Cox S, Frings D, Naughton F, Brown J, et al. E-cigarette support for smoking cessation: Identifying the effectiveness of intervention components in an on-line randomized optimization experiment. Addiction. 2023 Nov 1;118(11):2105–17.

30. Rethorst CD, Carmody TJ, Argenbright KE, Vazquez L, DeLuca T, Mayes TL, et al. The physical activity in cancer survivors (PACES) trial: a factorial randomized trial to optimize intervention for breast cancer survivors. Journal of Behavioral Medicine. 2024 Dec 1;47(6):1002–11.

31. Cleland CM, Gwadz M, Collins LM, Wilton L, Leonard NR, Ritchie AS, et al. Effects of Behavioral Intervention Components for African American/Black and Latino Persons Living with HIV with Non-suppressed Viral Load Levels: Results of an Optimization Trial. AIDS Behav. 2023 Nov;27(11):3695–712.

32. Edwardson CL, Biddle SJH, Clemes SA, Davies MJ, Dunstan DW, Eborall H, et al. Effectiveness of an intervention for reducing sitting time and improving health in office workers: three arm cluster randomised controlled trial. BMJ. 2022 Aug 17;378:e069288.

33. Andrews C, Whatley C, Smith M, Brayton EC, Simone S, Holmes AV. Quality-Improvement Effort to Reduce Hypothermia Among High-Risk Infants on a Mother-Infant Unit. Pediatrics. 2018 Mar;141(3):e20171214.

34. Forrest CB, Fiks AG, Bailey LC, Localio R, Grundmeier RW, Richards T, et al. Improving adherence to otitis media guidelines with clinical decision support and physician feedback. Pediatrics. 2013 Apr;131(4):e1071-1081.

35. Cox CE, Gallis JA, Olsen MK, Porter LS, Gremore T, Greeson JM, et al. Mobile Mindfulness Intervention for Psychological Distress Among Intensive Care Unit Survivors: A Randomized Clinical Trial. JAMA Internal Medicine. 2024 Jul 1;184(7):749–59.

36. Celano CM, Albanese AM, Millstein RA, Mastromauro CA, Chung WJ, Campbell KA, et al. Optimizing a Positive Psychology Intervention to Promote Health Behaviors After an Acute Coronary Syndrome: The Positive Emotions After Acute Coronary Events III (PEACE-III) Randomized Factorial Trial. Psychosom Med. 2018 Aug;80(6):526–34.

37. Sundström C, Peynenburg V, Chadwick C, Thiessen D, Wilhems A, Nugent M, et al. Optimizing internet-delivered cognitive behaviour therapy for alcohol misuse-a randomized factorial trial examining effects of a pre-treatment assessment interview and guidance. Addict Sci Clin Pract. 2022 Jul 23;17(1):37.

38. Baretta D, Amrein MA, Bäder C, Ruschetti GG, Rüttimann C, Del Rio Carral M, et al. Promoting Hand Hygiene During the COVID-19 Pandemic: Parallel Randomized Trial for the Optimization of the Soapp App. JMIR Mhealth Uhealth. 2023 Feb 3;11:e43241.

39. Cameron D, Morden JP, Canney P, Velikova G, Coleman R, Bartlett J, et al. Accelerated versus standard epirubicin followed by cyclophosphamide, methotrexate, and fluorouracil or capecitabine as adjuvant therapy for breast cancer in the randomised UK TACT2 trial (CRUK/05/19): a multicentre, phase 3, open-label, randomised, controlled trial. Lancet Oncol. 2017 Jul;18(7):929–45.

40. Erlinge D, Gurbel PA, James S, Lindahl TL, Svensson P, Ten Berg JM, et al. Prasugrel 5 mg in the very elderly attenuates platelet inhibition but maintains noninferiority to prasugrel 10 mg in nonelderly patients: the GENERATIONS trial, a pharmacodynamic and pharmacokinetic study in stable coronary artery disease patients. J Am Coll Cardiol. 2013 Aug 13;62(7):577–83.

41. Wyrick DL, Tanner AE, Milroy JJ, Guastaferro K, Bhandari S, Kugler KC, et al. itMatters: Optimization of an online intervention to prevent sexually transmitted infections in college students. J Am Coll Health. 2022 Jun;70(4):1212–22.

42. Zimmerman DL, Ruzicka M, Hebert P, Fergusson D, Touyz RM, Burns KD. Short Daily versus Conventional Hemodialysis for Hypertensive Patients: A Randomized Cross-Over Study. PLOS ONE. 2014 May 29;9(5):e97135.

43. Oliver DP, Washington K, Benson J, Kruse RL, Popejoy L, Liu J, et al. Access for Cancer Caregivers to Education and Support for Shared Decision Making (ACCESS) intervention: a cluster cross-over randomised clinical trial. BMJ Support Palliat Care. 2023 Mar 2;spcare-2022-004100.

44. O’Connor DA, Glasziou P, Maher CG, McCaffery KJ, Schram D, Maguire B, et al. Effect of an Individualized Audit and Feedback Intervention on Rates of Musculoskeletal Diagnostic Imaging Requests by Australian General Practitioners: A Randomized Clinical Trial. JAMA. 2022 Sep 6;328(9):850–60.

45. Rodríguez-Espinosa D, Cuadrado-Payán E, Rico N, Torra M, Fernández RM, Gómez M, et al. Comparative Effects of Acetate- and Citrate-Based Dialysates on Dialysis Dose and Protein-Bound Uremic Toxins in Hemodiafiltration Patients: Exploring the Impact of Calcium and Magnesium Concentrations. Toxins. 2024;16(10).

46. Vyas DP, Quinones-Cardona V, Gilfillan MA, Young ME, Pough KA, Carey AJ. Reduction of unnecessary antibiotic days in a level IV neonatal intensive care unit. Antimicrob Steward Healthc Epidemiol. 2022;2(1):e50.

47. Glinz D, Hurrell RF, Ouattara M, Zimmermann MB, Brittenham GM, Adiossan LG, et al. The effect of iron-fortified complementary food and intermittent preventive treatment of malaria on anaemia in 12- to 36-month-old children: a cluster-randomised controlled trial. Malaria Journal. 2015 Sep 17;14(1):347.

48. Roddy E, Ogollah RO, Oppong R, Zwierska I, Datta P, Hall A, et al. Optimising outcomes of exercise and corticosteroid injection in patients with subacromial pain (impingement) syndrome: a factorial randomised trial. Br J Sports Med. 2021 Mar;55(5):262–71.

49. Watkins E, Newbold A, Tester-Jones M, Collins LM, Mostazir M. Investigation of Active Ingredients Within Internet-Delivered Cognitive Behavioral Therapy for Depression: A Randomized Optimization Trial. JAMA Psychiatry. 2023 Sep 1;80(9):942–51.

50. Geng EH, Odeny TA, Montoya LM, Iguna S, Kulzer JL, Adhiambo HF, et al. Adaptive Strategies for Retention in Care among Persons Living with HIV. NEJM Evid. 2023 Apr;2(4).

51. Pino FA, Sam KJ, Wood SL, Tafreshi PA, Parks SL, Bell PA, et al. Increasing Compliance with a New Interunit Handoff Process: A Quality Improvement Project. Pediatr Qual Saf. 2019 Jun;4(3):e180.

52. Semitala FC, Kadota JL, Musinguzi A, Welishe F, Nakitende A, Akello L, et al. Comparison of 3 optimized delivery strategies for completion of isoniazid-rifapentine (3HP) for tuberculosis prevention among people living with HIV in Uganda: A single-center randomized trial. PLoS Med. 2024 Feb;21(2):e1004356.

53. Patti G, Tomai F, Melfi R, Ricottini E, Macrì M, Sedati P, et al. Strategies of clopidogrel load and atorvastatin reload to prevent ischemic cerebral events in patients undergoing protected carotid stenting. Results of the randomized ARMYDA-9 CAROTID (Clopidogrel and Atorvastatin Treatment During Carotid Artery Stenting) study. J Am Coll Cardiol. 2013 Apr 2;61(13):1379–87.

54. Weise H, Zenner B, Schmiedchen B, Benning L, Bulitta M, Schmitz D, et al. The Effect of an App-Based Home Exercise Program on Self-reported Pain Intensity in Unspecific and Degenerative Back Pain: Pragmatic Open-label Randomized Controlled Trial. J Med Internet Res. 2022 Oct 28;24(10):e41899.

55. Seddik H, Benass J, Berrag S, Sair A, Berraida R, Boutallaka H. Optimized sequential therapy vs 10- and 14-d concomitant therapy for eradicating Helicobacter pylori: A randomized clinical trial. World J Gastroenterol. 2024 Feb 14;30(6):556–64.

56. Tombor I, Beard E, Brown J, Shahab L, Michie S, West R. Randomized factorial experiment of components of the SmokeFree Baby smartphone application to aid smoking cessation in pregnancy. Transl Behav Med. 2019 Jul 16;9(4):583–93.

57. Graat I, van Rooijen G, Prinsen J, Bergfeld I, Figee M, Denys D, et al. Cyclic versus continuous deep brain stimulation in patients with obsessive compulsive disorder: A randomized controlled trial. Brain Stimul. 2023 Feb;16(1):82–7.

58. Forrester JA, Starr N, Negussie T, Schaps D, Adem M, Alemu S, et al. Clean Cut (adaptive, multimodal surgical infection prevention programme) for low-resource settings: a prospective quality improvement study. Br J Surg. 2021 Jun 22;108(6):727–34.

59. Stevens J, Trimboli A, Samios P, Steele N, Welch S, Thompson P, et al. A sustainable method to reduce postoperative oxycodone discharge prescribing in a metropolitan tertiary referral hospital. Anaesthesia. 2019 Mar;74(3):292–9.

60. Dekker J, Van HL, Hendriksen M, Koelen J, Schoevers RA, Kool S, et al. What is the best sequential treatment strategy in the treatment of depression? Adding pharmacotherapy to psychotherapy or vice versa? Psychother Psychosom. 2013;82(2):89–98.

61. Kaur J, Singh S, Vij J. Optimization of Efficacy of Core Strengthening Exercise Protocols on Patients Suffering from Diabetes Mellitus. Romanian Journal of Diabetes Nutrition and Metabolic Diseases. 2018 Mar 1;25:23–36.

62. Kaur J, Singh S, Vij J. Optimization of Aerobic Exercise Protocols in Diabetes Mellitus: A Randomized Trial. Romanian Journal of Diabetes Nutrition and Metabolic Diseases. 2017 Dec 15;24.

63. Bigna JJR, Noubiap JJN, Kouanfack C, Plottel CS, Koulla-Shiro S. Effect of mobile phone reminders on follow-up medical care of children exposed to or infected with HIV in Cameroon (MORE CARE): a multicentre, single-blind, factorial, randomised controlled trial. Lancet Infect Dis. 2014 Jul;14(7):600–8.

64. Pernica JM, Arscott-Mills T, Steenhoff AP, Mokomane M, Moorad B, Bapabi M, et al. Optimising the management of childhood acute diarrhoeal disease using a rapid test-and- treat strategy and/or Lactobacillus reuteri DSM 17938: a multicentre, randomised, controlled, factorial trial in Botswana. BMJ Glob Health. 2022 Apr;7(4).

65. McClure JB, Shortreed SM, Bogart A, Derry H, Riggs K, St John J, et al. The effect of program design on engagement with an internet-based smoking intervention: randomized factorial trial. J Med Internet Res. 2013 Mar 25;15(3):e69.

66. Abraham J, Kupfer R, Behncke A, Berger-Höger B, Icks A, Haastert B, et al. Implementation of a multicomponent intervention to prevent physical restraints in nursing homes (IMPRINT): A pragmatic cluster randomized controlled trial. Int J Nurs Stud. 2019 Aug;96:27–34.

67. Krebs JD, Weatherall M, Corley B, Wiltshire E, McTavish L. Optimizing the management of hypoglycaemia in individuals with type 2 diabetes: A randomized crossover comparison of a weight-based protocol compared with two fixed-dose glucose regimens. Diabetes Obes Metab. 2018 May;20(5):1256–61.

68. Fortney JC, Pyne JM, Kimbrell TA, Hudson TJ, Robinson DE, Schneider R, et al. Telemedicine-based collaborative care for posttraumatic stress disorder: a randomized clinical trial. JAMA Psychiatry. 2015 Jan;72(1):58–67.

69. Fortney JC, Bauer AM, Cerimele JM, Pyne JM, Pfeiffer P, Heagerty PJ, et al. Comparison of Teleintegrated Care and Telereferral Care for Treating Complex Psychiatric Disorders in Primary Care: A Pragmatic Randomized Comparative Effectiveness Trial. JAMA Psychiatry. 2021 Nov 1;78(11):1189–99.

70. Fortney JC, Pyne JM, Mouden SB, Mittal D, Hudson TJ, Schroeder GW, et al. Practice-based versus telemedicine-based collaborative care for depression in rural federally qualified health centers: a pragmatic randomized comparative effectiveness trial. Am J Psychiatry. 2013 Apr;170(4):414–25.

71. Tilburt JC, Zahrieh D, Pacyna JE, Petereit DG, Kaur JS, Rapkin BD, et al. Decision aids for localized prostate cancer in diverse minority men: Primary outcome results from a multicenter cancer care delivery trial (Alliance A191402CD). Cancer. 2022 Mar 15;128(6):1242–51.

72. Emery JD, Gray V, Walter FM, Cheetham S, Croager EJ, Slevin T, et al. The Improving Rural Cancer Outcomes Trial: a cluster-randomised controlled trial of a complex intervention to reduce time to diagnosis in rural cancer patients in Western Australia. Br J Cancer. 2017 Nov 7;117(10):1459–69.

73. Bisson JI, Ariti C, Cullen K, Kitchiner N, Lewis C, Roberts NP, et al. Guided, internet based, cognitive behavioural therapy for post-traumatic stress disorder: pragmatic, multicentre, randomised controlled non-inferiority trial (RAPID). BMJ. 2022 Jun 16;377:e069405.

74. Schmitz JM, Stotts AL, Vujanovic AA, Yoon JH, Webber HE, Lane SD, et al. Contingency management plus acceptance and commitment therapy for initial cocaine abstinence: Results of a sequential multiple assignment randomized trial (SMART). Drug Alcohol Depend. 2024 Mar 1;256:111078.

75. Pa J, Goodson W, Bloch A, King AC, Yaffe K, Barnes DE. Effect of exercise and cognitive activity on self-reported sleep quality in community-dwelling older adults with cognitive complaints: a randomized controlled trial. J Am Geriatr Soc. 2014 Dec;62(12):2319–26.

76. Fritz JM, Sharpe J, Greene T, Lane E, Hadizadeh M, McFadden M, et al. Optimization of Spinal Manipulative Therapy Protocols: A Factorial Randomized Trial Within a Multiphase Optimization Framework. J Pain. 2021 Jun;22(6):655–68.

77. Foster JM, Usherwood T, Smith L, Sawyer SM, Xuan W, Rand CS, et al. Inhaler reminders improve adherence with controller treatment in primary care patients with asthma. J Allergy Clin Immunol. 2014 Dec;134(6):1260-1268.e3.

78. Rosenstock J, Bergenstal RM, Blevins TC, Morrow LA, Prince MJ, Qu Y, et al. Better glycemic control and weight loss with the novel long-acting basal insulin LY2605541 compared with insulin glargine in type 1 diabetes: a randomized, crossover study. Diabetes Care. 2013 Mar;36(3):522–8.

79. Brown JC, Sturgeon K, Sarwer DB, Troxel AB, DeMichele AM, Denlinger CS, et al. The effects of exercise and diet on oxidative stress and telomere length in breast cancer survivors. Breast Cancer Res Treat. 2023 May;199(1):109–17.

80. Su KC, Kou YR, Lin FC, Wu CH, Feng JY, Huang SF, et al. A simplified prevention bundle with dual hand hygiene audit reduces early-onset ventilator-associated pneumonia in cardiovascular surgery units: An interrupted time-series analysis. PLoS One. 2017;12(8):e0182252.

81. Fizazi K, Foulon S, Carles J, Roubaud G, McDermott R, Fléchon A, et al. Abiraterone plus prednisone added to androgen deprivation therapy and docetaxel in de novo metastatic castration-sensitive prostate cancer (PEACE-1): a multicentre, open-label, randomised, phase 3 study with a 2 × 2 factorial design. Lancet. 2022 Apr 30;399(10336):1695–707.

82. Tao KFM, Moreira T de C, Jayakody DMP, Swanepoel DW, Brennan-Jones CG, Coetzee L, et al. Teleaudiology hearing aid fitting follow-up consultations for adults: single blinded crossover randomised control trial and cohort studies. Int J Audiol. 2021 Apr;60(sup1):S49–60.

83. Manning K, Senekal M, Harbron J. Group-based intervention in a primary healthcare setting was more effective for weight loss than usual care. Health SA. 2019;24:1172.

84. Rulison KL, Milroy JJ, Wyrick DL. A randomized iterative approach to optimizing an online substance use intervention for collegiate athletes. Transl Behav Med. 2022 Jan 18;12(1).

85. Schwartz KL, Shuldiner J, Langford BJ, Brown KA, Schultz SE, Leung V, et al. Mailed feedback to primary care physicians on antibiotic prescribing for patients aged 65 years and older: pragmatic, factorial randomised controlled trial. BMJ. 2024 Jun 5;385:e079329.

86. Richter KP, Shireman TI, Ellerbeck EF, Cupertino AP, Catley D, Cox LS, et al. Comparative and cost effectiveness of telemedicine versus telephone counseling for smoking cessation. J Med Internet Res. 2015 May 8;17(5):e113.

87. Kemmeren LL, van Schaik A, Draisma S, Kleiboer A, Riper H, Smit JH. Effectiveness of Blended Cognitive Behavioral Therapy Versus Treatment as Usual for Depression in Routine Specialized Mental Healthcare: E-COMPARED Trial in the Netherlands. Cognitive Therapy and Research. 2023 Jun 1;47(3):386–98.

88. Bell L, Garnett C, Bao Y, Cheng Z, Qian T, Perski O, et al. How Notifications Affect Engagement With a Behavior Change App: Results From a Micro-Randomized Trial. JMIR Mhealth Uhealth. 2023 Jun 9;11:e38342.

89. Eberly LA, Tennison A, Mays D, Hsu CY, Yang CT, Benally E, et al. Telephone-Based Guideline-Directed Medical Therapy Optimization in Navajo Nation: The Hózhó Randomized Clinical Trial. JAMA Intern Med. 2024 Jun 1;184(6):681–90.

90. Fisher L, Hessler D, Glasgow RE, Arean PA, Masharani U, Naranjo D, et al. REDEEM: a pragmatic trial to reduce diabetes distress. Diabetes Care. 2013 Sep;36(9):2551–8.

91. Morrison L, Moss-Morris R, Michie S, Yardley L. Optimizing engagement with Internet-based health behaviour change interventions: comparison of self-assessment with and without tailored feedback using a mixed methods approach. Br J Health Psychol. 2014 Nov;19(4):839–55.

92. Rindner L, Nordeman L, Strömme G, Hange D, Gunnarsson R, Rembeck G. Effect of group education and person-centered support in primary health care on mental health and quality of life in women aged 45-60 years with symptoms commonly associated with stress: a randomized controlled trial. BMC Womens Health. 2023 Mar 24;23(1):128.

93. Windsor LC, Benoit E, Lee C, Jemal A, Kugler K, Smith DC, et al. Critical Dialogue and Capacity-Building Projects Reduced Alcohol and Substance Use in a Randomized Clinical Trial Among Formerly Incarcerated Men. Subst Use Misuse. 2024;59(11):1574–85.

94. Bilello LA, Livingood WC, Lukens-Bull K, Smotherman C, Choe U. Texting Test Results Reduces the Time to Treatment for Sexually Transmitted Infections. J Public Health Manag Pract. 2019 Apr;25(2):165–70.

95. Bell LK, Morgillo S, Zarnowiecki D, Gardner C, Leemaqz S, Arguelles J, et al. Development of an initiatives package to increase children’s vegetable intake in long day care centres using the Multiphase Optimisation Strategy (MOST) randomised factorial experiment. Public Health Nutr. 2023 Dec;26(12):3062–75.

96. Wagner LI, Tooze JA, Hall DL, Levine BJ, Beaumont J, Duffecy J, et al. Targeted eHealth Intervention to Reduce Breast Cancer Survivors’ Fear of Recurrence: Results From the FoRtitude Randomized Trial. J Natl Cancer Inst. 2021 Nov 2;113(11):1495–505.

97. Abreu M, Tumyan A, Elhassan A, Peicher K, Papacostea O, Dimachkie P, et al. A randomized trial comparing the efficacy and safety of treating patients with type 2 diabetes and highly elevated HbA1c levels with basal-bolus insulin or a glucagon-like peptide-1 receptor agonist plus basal insulin: The SIMPLE study. Diabetes Obes Metab. 2019 Sep;21(9):2133–41.

98. de Gier M, Beckerman H, Twisk J, Knoop H, de Groot V. Blended versus face-to-face cognitive behavioural therapy for severe fatigue in patients with multiple sclerosis: A non-inferiority RCT. Mult Scler. 2023 Sep;29(10):1316–26.

99. Neuman MD, Feng R, Shukla AS, Han X, Horan AD, Whatley K, et al. Strategies to Limit Benzodiazepine Use in Anesthesia for Older Adults: A Randomized Clinical Trial. JAMA Netw Open. 2024 Oct 1;7(10):e2442207.

100. Sakata M, Toyomoto R, Yoshida K, Luo Y, Nakagami Y, Uwatoko T, et al. Components of smartphone cognitive-behavioural therapy for subthreshold depression among 1093 university students: a factorial trial. Evid Based Ment Health. 2022 Dec;25(e1):e18–25.

101. Berg M, Rozental A, de Brun Mangs J, Näsman M, Strömberg K, Viberg L, et al. The Role of Learning Support and Chat-Sessions in Guided Internet-Based Cognitive Behavioral Therapy for Adolescents With Anxiety: A Factorial Design Study. Frontiers in Psychiatry [Internet]. 2020;Volume 11-2020. Available from: https://www.frontiersin.org/journals/psychiatry/articles/10.3389/fpsyt.2020.00503

102. Shulman M, Greiner MG, Tafessu HM, Opara O, Ohrtman K, Potter K, et al. Rapid Initiation of Injection Naltrexone for Opioid Use Disorder: A Stepped-Wedge Cluster Randomized Clinical Trial. JAMA Netw Open. 2024 May 1;7(5):e249744.

103. Gilkey MB, Heisler-MacKinnon J, Boynton MH, Calo WA, Moss JL, Brewer NT. Impact of Brief Quality Improvement Coaching on Adolescent HPV Vaccination Coverage: A Pragmatic Cluster Randomized Trial. Cancer Epidemiol Biomarkers Prev. 2023 Jul 5;32(7):957–62.

104. Heisler M, Burgess J, Cass J, Chardos JF, Guirguis AB, Strohecker LA, et al. Evaluating the Effectiveness of Diabetes Shared Medical Appointments (SMAs) as Implemented in Five Veterans Affairs Health Systems: a Multi-site Cluster Randomized Pragmatic Trial. J Gen Intern Med. 2021 Jun;36(6):1648–55.

105. Abdel-Fattah M, Cooper D, Davidson T, Kilonzo M, Hossain M, Boyers D, et al. Single-Incision Mini-Slings for Stress Urinary Incontinence in Women. N Engl J Med. 2022 Mar 31;386(13):1230–43.

106. Abdel-Ghaffar ME, Ismail SA, Ismail RA, Abdelrahman MM, Abuelnaga ME. Comparison Between Two Volumes of 70% Alcohol in Single Injection Ultrasound-Guided Celiac Plexus Neurolysis: A Randomized Controlled Trial. Pain Physician. 2022 May;25(3):293–303.

107. Köhle N, Drossaert CHC, Ten Klooster PM, Schreurs KMG, Hagedoorn M, Van Uden-Kraan CF, et al. Web-based self-help intervention for partners of cancer patients based on acceptance and commitment therapy and self-compassion training: a randomized controlled trial with automated versus personal feedback. Support Care Cancer. 2021 Sep;29(9):5115–25.

108. Sherwood NE, Crain AL, Seburg EM, Butryn ML, Forman EM, Crane MM, et al. BestFIT Sequential Multiple Assignment Randomized Trial Results: A SMART Approach to Developing Individualized Weight Loss Treatment Sequences. Ann Behav Med. 2022 Mar 1;56(3):291–304.

109. Whitesell NR, Mousseau AC, Keane EM, Asdigian NL, Tuitt N, Morse B, et al. Integrating Community-Engagement and a Multiphase Optimization Strategy Framework: Adapting Substance Use Prevention for American Indian Families. Prev Sci. 2019 Oct;20(7):1136–46.

110. Jennings N, Gardner G, O’Reilly G, Mitra B. Evaluating emergency nurse practitioner service effectiveness on achieving timely analgesia: a pragmatic randomized controlled trial. Acad Emerg Med. 2015 Jun;22(6):676–84.

111. Thiruchelvam N, Landauro MH, Biardeau X, Rovsing C, Hahn M, Nascimento OF do, et al. Improved emptying performance with a new micro-hole zone catheter in adult male intermittent catheter users: A comparative multi-center randomized controlled cross-over study. Neurourol Urodyn. 2024 Feb;43(2):464–78.

112. Ivers NM, Schwalm JD, Bouck Z, McCready T, Taljaard M, Grace SL, et al. Interventions supporting long term adherence and decreasing cardiovascular events after myocardial infarction (ISLAND): pragmatic randomised controlled trial. BMJ. 2020 Jun 10;369:m1731.

113. Manzi O, Ogbuagu O. SEQUENTIAL LOW COST INTERVENTIONS DOUBLE HAND HYGIENE RATES AMONG MEDICAL TEAMS IN A RESOURCE LIMITED SETTING. RESULTS OF A HAND HYGIENE QUALITY IMPROVEMENT PROJECT CONDUCTED AT UNIVERSITY TEACHING HOSPITAL OF KIGALI (CHUK), KIGALI, RWANDA. East Afr Med J. 2014 Feb;91(2):44–9.

114. Essien O, Otu A, Umoh V, Enang O, Hicks JP, Walley J. Intensive Patient Education Improves Glycaemic Control in Diabetes Compared to Conventional Education: A Randomised Controlled Trial in a Nigerian Tertiary Care Hospital. PLoS One. 2017;12(1):e0168835.

115. Bur OT, Krieger T, Moritz S, Klein JP, Berger T. Optimizing the context of support of web-based self-help in individuals with mild to moderate depressive symptoms: A randomized full factorial trial. Behav Res Ther. 2022 May;152:104070.

116. Mimoz O, Lucet JC, Kerforne T, Pascal J, Souweine B, Goudet V, et al. Skin antisepsis with chlorhexidine-alcohol versus povidone iodine-alcohol, with and without skin scrubbing, for prevention of intravascular-catheter-related infection (CLEAN): an open-label, multicentre, randomised, controlled, two-by-two factorial trial. Lancet. 2015 Nov 21;386(10008):2069–77.

117. Ramnarayan P, Richards-Belle A, Drikite L, Saull M, Orzechowska I, Darnell R, et al. Effect of High-Flow Nasal Cannula Therapy vs Continuous Positive Airway Pressure Therapy on Liberation From Respiratory Support in Acutely Ill Children Admitted to Pediatric Critical Care Units: A Randomized Clinical Trial. JAMA. 2022 Jul 12;328(2):162–72.

118. Lavoie PM, Stritzke A, Ting J, Jabr M, Jain A, Kwan E, et al. A Randomized Controlled Trial of the Use of Oral Glucose with or without Gentle Facilitated Tucking of Infants during Neonatal Echocardiography. PLoS One. 2015;10(10):e0141015.

119. Martínez-Ibáñez P, Marco-Moreno I, Peiró S, Martínez-Ibáñez L, Barreira-Franch I, Bellot-Pujalte L, et al. Home Blood Pressure Self-monitoring plus Self-titration of Antihypertensive Medication for Poorly Controlled Hypertension in Primary Care: the ADAMPA Randomized Clinical Trial. J Gen Intern Med. 2023 Jan;38(1):81–9.

120. Łakuta P. A Factorial Randomized Controlled Trial of Implementation-Intention-Based Self-Affirmation Interventions: Findings on Depression, Anxiety, and Well-being in Adults With Psoriasis. Front Psychiatry. 2022;13:795055.

121. Anderson P, Bendtsen P, Spak F, Reynolds J, Drummond C, Segura L, et al. Improving the delivery of brief interventions for heavy drinking in primary health care: outcome results of the Optimizing Delivery of Health Care Intervention (ODHIN) five-country cluster randomized factorial trial. Addiction. 2016 Nov;111(11):1935–45.

122. Pirolli P, Mohan S, Venkatakrishnan A, Nelson L, Silva M, Springer A. Implementation Intention and Reminder Effects on Behavior Change in a Mobile Health System: A Predictive Cognitive Model. J Med Internet Res. 2017 Nov 30;19(11):e397.

123. Szilagyi P, Albertin C, Gurfinkel D, Beaty B, Zhou X, Vangala S, et al. Effect of State Immunization Information System Centralized Reminder and Recall on HPV Vaccination Rates. Pediatrics. 2020 May;145(5).

124. Denig P, Schuling J, Haaijer-Ruskamp F, Voorham J. Effects of a patient oriented decision aid for prioritising treatment goals in diabetes: pragmatic randomised controlled trial. BMJ. 2014 Sep 25;349:g5651.

125. Meena P, Kumar A, Sodhi MK, Grover S, Tania. Quality Improvement Initiative To Improve Breastfeeding Rates And Monitoring Of Full Term Neonates In The First Hour Of Life. International Journal of Life Sciences, Biotechnology and Pharma Research. 2024 Nov;13(11).

126. Klasnja P, Smith S, Seewald NJ, Lee A, Hall K, Luers B, et al. Efficacy of Contextually Tailored Suggestions for Physical Activity: A Micro-randomized Optimization Trial of HeartSteps. Ann Behav Med. 2019 May 3;53(6):573–82.

127. Palmer R, Dimairo M, Cooper C, Enderby P, Brady M, Bowen A, et al. Self-managed, computerised speech and language therapy for patients with chronic aphasia post-stroke compared with usual care or attention control (Big CACTUS): a multicentre, single-blinded, randomised controlled trial. Lancet Neurol. 2019 Sep;18(9):821–33.

128. Holland R, Maskrey V, Swift L, Notley C, Robinson A, Nagar J, et al. Treatment retention, drug use and social functioning outcomes in those receiving 3 months versus 1 month of supervised opioid maintenance treatment. Results from the Super C randomized controlled trial. Addiction. 2014 Apr;109(4):596–607.

129. Fortuna RJ, Idris A, Winters P, Humiston SG, Scofield S, Hendren S, et al. Get screened: a randomized trial of the incremental benefits of reminders, recall, and outreach on cancer screening. J Gen Intern Med. 2014 Jan;29(1):90–7.

130. Collado-Borrell R, Escudero-Vilaplana V, Ribed A, Gonzalez-Anleo C, Martin-Conde M, Romero-Jimenez R, et al. Effect of a Mobile App for the Pharmacotherapeutic Follow-Up of Patients With Cancer on Their Health Outcomes: Quasi-Experimental Study. JMIR Mhealth Uhealth. 2020 Oct 16;8(10):e20480.

131. James RD, Glynne-Jones R, Meadows HM, Cunningham D, Myint AS, Saunders MP, et al. Mitomycin or cisplatin chemoradiation with or without maintenance chemotherapy for treatment of squamous-cell carcinoma of the anus (ACT II): a randomised, phase 3, open-label, 2 × 2 factorial trial. Lancet Oncol. 2013 May;14(6):516–24.

132. Prinz RJ, Metzler CW, Sanders MR, Rusby JC, Cai C. Online-delivered parenting intervention for young children with disruptive behavior problems: a noninferiority trial focused on child and parent outcomes. J Child Psychol Psychiatry. 2022 Feb;63(2):199–209.

133. Uthaya S, Liu X, Babalis D, Doré CJ, Warwick J, Bell J, et al. Nutritional Evaluation and Optimisation in Neonates: a randomized, double-blind controlled trial of amino acid regimen and intravenous lipid composition in preterm parenteral nutrition. Am J Clin Nutr. 2016 Jun;103(6):1443–52.

134. Shukla S, Cortez J, Renfro B, Makker K, Timmons C, Nandula PS, et al. Charge Nurses Taking Charge, Challenging the Culture of Culture-Negative Sepsis, and Preventing Central-Line Infections to Reduce NICU Antibiotic Usage. Am J Perinatol. 2022 Jun;39(8):861–8.

135. Hayes SC, Rye S, Disipio T, Yates P, Bashford J, Pyke C, et al. Exercise for health: a randomized, controlled trial evaluating the impact of a pragmatic, translational exercise intervention on the quality of life, function and treatment-related side effects following breast cancer. Breast Cancer Res Treat. 2013 Jan;137(1):175–86.

136. Parekh S, King D, Boyle FM, Vandelanotte C. Randomized controlled trial of a computer-tailored multiple health behaviour intervention in general practice: 12-month follow-up results. Int J Behav Nutr Phys Act. 2014 Mar 19;11(1):41.

137. Kalkhoran S, Inman EM, Kelley JHK, Ashburner JM, Rigotti NA. Proactive Population Health Strategy to Offer Tobacco Dependence Treatment to Smokers in a Primary Care Practice Network. J Gen Intern Med. 2019 Aug;34(8):1571–7.

138. Cutrona SL, Golden JG, Goff SL, Ogarek J, Barton B, Fisher L, et al. Improving Rates of Outpatient Influenza Vaccination Through EHR Portal Messages and Interactive Automated Calls: A Randomized Controlled Trial. J Gen Intern Med. 2018 May;33(5):659–67.

139. Cullen SM, Osorio SN, Abramson EA, Kyvelos E. Improving Caregiver Understanding of Liquid Acetaminophen Administration at Primary Care Visits. Pediatrics. 2022 Aug 1;150(2):e2021054807.

140. Himelhoch S, Kelly D, deFilippi C, Taylor G, Bennett M, Medoff D, et al. Optimizing behavioral and pharmacological smoking cessation interventions among people with HIV. AIDS. 2024 Apr 1;38(5):669–78.

141. Ramey SL, DeLuca SC, Stevenson RD, Conaway M, Darragh AR, Lo W. Constraint-Induced Movement Therapy for Cerebral Palsy: A Randomized Trial. Pediatrics. 2021 Nov;148(5).

142. AlHarthy SH, Al-MoundhrI M, Al-Mahmoodi W, Ibrahim R, Ayaad O, Al Baimani K. Referral Process Enhancement: Innovative Approaches and Best Practices. Asian Pac J Cancer Prev. 2024 May 1;25(5):1691–8.

143. AlHarthy SH, Ayaad O, Al Mashari AAA, AlBalushi MA, Ibrahim R, Bait Nasib MH, et al. Improving Care Continuity in Oncology Settings: A Lean Management Approach to Minimize Discharges Without Follow-Up Appointments. Asian Pac J Cancer Prev. 2024 Apr 1;25(4):1293–300.

144. Mehta SJ, Volpp KG, Troxel AB, Day SC, Lim R, Marcus N, et al. Electronic Pill Bottles or Bidirectional Text Messaging to Improve Hypertension Medication Adherence (Way 2 Text): a Randomized Clinical Trial. J Gen Intern Med. 2019 Nov;34(11):2397–404.

145. Bi SY, Yu YH, Li C, Xu P, Xu HY, Li JH, et al. A standardized implementation of multicenter quality improvement program of very low birth weight newborns could significantly reduce admission hypothermia and improve outcomes. BMC Pediatr. 2022 May 14;22(1):281.

146. Gilbody S, Littlewood E, Hewitt C, Brierley G, Tharmanathan P, Araya R, et al. Computerised cognitive behaviour therapy (cCBT) as treatment for depression in primary care (REEACT trial): large scale pragmatic randomised controlled trial. BMJ. 2015 Nov 11;351:h5627.

147. Stanworth S, Walwyn R, Grant-Casey J, Hartley S, Moreau L, Lorencatto F, et al. Effectiveness of Enhanced Performance Feedback on Appropriate Use of Blood Transfusions: A Comparison of 2 Cluster Randomized Trials. Jama Network Open. 2022 Feb 24;5.

148. Boyce SP, Nyangara F, Kamunyori J. A mixed-methods quasi-experimental evaluation of a mobile health application and quality of care in the integrated community case management program in Malawi. J Glob Health. 2019 Jun;9(1):010811.

149. Scalvini S, Zanelli E, Comini L, Dalla Tomba M, Troise G, Febo O, et al. Home-based versus in-hospital cardiac rehabilitation after cardiac surgery: a nonrandomized controlled study. Phys Ther. 2013 Aug;93(8):1073–83.

150. Phillips SM, Penedo FJ, Collins LM, Solk P, Siddique J, Song J, et al. Optimization of a technology-supported physical activity promotion intervention for breast cancer survivors: Results from Fit2Thrive. Cancer. 2022 Mar 1;128(5):1122–32.

151. Tesfaye S, Sloan G, Petrie J, White D, Bradburn M, Julious S, et al. Comparison of amitriptyline supplemented with pregabalin, pregabalin supplemented with amitriptyline, and duloxetine supplemented with pregabalin for the treatment of diabetic peripheral neuropathic pain (OPTION-DM): a multicentre, double-blind, randomised crossover trial. The Lancet. 2022 Aug 27;400(10353):680–90.

152. Green SMC, Hall LH, French DP, Rousseau N, Parbutt C, Walwyn R, et al. Optimization of an Information Leaflet to Influence Medication Beliefs in Women With Breast Cancer: A Randomized Factorial Experiment. Ann Behav Med. 2023 Oct 16;57(11):988–1000.

153. Kerrigan SG, Forman EM, Williams D, Patel M, Loyka C, Zhang F, et al. Project Step: A Randomized Controlled Trial Investigating the Effects of Frequent Feedback and Contingent Incentives on Physical Activity. J Phys Act Health. 2021 Mar 1;18(3):247–53.

154. Bernstein SL, Dziura J, Weiss J, Brooks AH, Miller T, Vickerman KA, et al. Successful Optimization of Tobacco Dependence Treatment in the Emergency Department: A Randomized Controlled Trial Using the Multiphase Optimization Strategy. Ann Emerg Med. 2023 Feb;81(2):209–21.

155. Fu SS, Rothman AJ, Vock DM, Lindgren BR, Almirall D, Begnaud A, et al. Optimizing Longitudinal Tobacco Cessation Treatment in Lung Cancer Screening: A Sequential, Multiple Assignment, Randomized Trial. JAMA Network Open. 2023 Aug 24;6(8):e2329903–e2329903.

156. Little SA, Leelarathna L, Walkinshaw E, Tan HK, Chapple O, Lubina-Solomon A, et al. Recovery of hypoglycemia awareness in long-standing type 1 diabetes: a multicenter 2 × 2 factorial randomized controlled trial comparing insulin pump with multiple daily injections and continuous with conventional glucose self-monitoring (HypoCOMPaSS). Diabetes Care. 2014 Aug;37(8):2114–22.

157. Kadura S, Eisner L, Lopa SH, Poulakis A, Mesmer H, Willnow N, et al. Nudging towards Sleep-Friendly Health Care: A Multifaceted Approach on Reducing Unnecessary Overnight Interventions. Appl Clin Inform. 2024 Oct;15(5):1025–39.

158. Kripalani S, Chen G, Ciampa P, Theobald C, Cao A, McBride M, et al. A transition care coordinator model reduces hospital readmissions and costs. Contemp Clin Trials. 2019 Jun;81:55–61.

159. Akechi T, Furukawa TA, Noma H, Iwata H, Toyama T, Higaki K, et al. Optimizing smartphone psychotherapy for depressive symptoms in patients with cancer: Multiphase optimization strategy using a decentralized multicenter randomized clinical trial (J-SUPPORT 2001 Study). Psychiatry Clin Neurosci. 2024 Jun;78(6):353–61.

160. Kortekangas T, Haapasalo H, Flinkkilä T, Ohtonen P, Nortunen S, Laine HJ, et al. Three week versus six week immobilisation for stable Weber B type ankle fractures: randomised, multicentre, non-inferiority clinical trial. BMJ. 2019 Jan 23;364:k5432.

161. Zeng T, Yuan H, Ren J, Li Y, Hou J, Du L, et al. A Pragmatic Study of Basal and Mid-Mixture Insulins as Starter Insulins in Chinese Patients With Type 2 Diabetes: Observations From Long-Term, Real-World Experience. Diabetes Ther. 2021 Mar;12(3):931–41.

162. Colbourn T, Nambiar B, Bondo A, Makwenda C, Tsetekani E, Makonda-Ridley A, et al. Effects of quality improvement in health facilities and community mobilization through women’s groups on maternal, neonatal and perinatal mortality in three districts of Malawi: MaiKhanda, a cluster randomized controlled effectiveness trial. Int Health. 2013 Sep;5(3):180–95.

163. Morgenthaler TI, Kuzniar TJ, Wolfe LF, Willes L, McLain WC 3rd, Goldberg R. The complex sleep apnea resolution study: a prospective randomized controlled trial of continuous positive airway pressure versus adaptive servoventilation therapy. Sleep. 2014 May 1;37(5):927–34.

164. Du T, Chidambaran V, Kara ST, Frazier M, Anadio J, Girten S, et al. Timely completion of spinal fusion: A multidisciplinary quality improvement initiative to improve operating room efficiency. Paediatr Anaesth. 2022 Aug;32(8):926–36.

165. Ow TW, Sukocheva O, Bampton P, Iyngkaran G, Rayner CK, Tse E. Improving Concordance Between Clinicians With Australian Guidelines for Bowel Cancer Prevention Using a Digital Application: Randomized Controlled Crossover Study. JMIR Cancer. 2024 Feb 22;10:e46625.

166. Chakravarthy U, Harding SP, Rogers CA, Downes S, Lotery AJ, Dakin HA, et al. A randomised controlled trial to assess the clinical effectiveness and cost-effectiveness of alternative treatments to Inhibit VEGF in Age-related choroidal Neovascularisation (IVAN). Health Technol Assess. 2015 Oct;19(78):1–298.

167. Fontil V, Modrow MF, Cooper-DeHoff RM, Wozniak G, Rakotz M, Todd J, et al. Improvement in Blood Pressure Control in Safety Net Clinics Receiving 2 Versions of a Scalable Quality Improvement Intervention: BP MAP A Pragmatic Cluster Randomized Trial. J Am Heart Assoc. 2023 Feb 7;12(3):e024975.

168. Ramineni V, Millroth P, Iyadurai L, Jaki T, Kingslake J, Highfield J, et al. Treating intrusive memories after trauma in healthcare workers: a Bayesian adaptive randomised trial developing an imagery-competing task intervention. Mol Psychiatry. 2023 Jul;28(7):2985–94.

169. Yakovchenko V, DeSotto K, Drainoni ML, Lukesh W, Miller DR, Park A, et al. Using Lean-Facilitation to Improve Quality of Hepatitis C Testing in Primary Care. J Gen Intern Med. 2021 Feb;36(2):349–57.

170. Orcel V, Banh L, Bastuji-Garin S, Renard V, Boutin E, Gouja A, et al. Effectiveness of comprehensive geriatric assessment adapted to primary care when provided by a nurse or a general practitioner: the CEpiA cluster-randomised trial. BMC Med. 2024 Sep 27;22(1):414.

171. Stergiopoulos V, Schuler A, Nisenbaum R, deRuiter W, Guimond T, Wasylenki D, et al. The effectiveness of an integrated collaborative care model vs. a shifted outpatient collaborative care model on community functioning, residential stability, and health service use among homeless adults with mental illness: a quasi-experimental study. BMC Health Serv Res. 2015 Aug 28;15:348.

172. van Vugt VA, van der Wouden JC, Essery R, Yardley L, Twisk JWR, van der Horst HE, et al. Internet based vestibular rehabilitation with and without physiotherapy support for adults aged 50 and older with a chronic vestibular syndrome in general practice: three armed randomised controlled trial. BMJ. 2019 Nov 5;367:l5922.

173. Dauphinot V, Boublay N, Moutet C, Achi S, Bathsavanis A, Krolak-Salmon P. Comparison of Instrumental Activities of Daily Living assessment by face-to-face or telephone interviews: a randomized, crossover study. Alzheimer’s Research & Therapy. 2020 Mar 13;12(1):24.

174. Han WM, Koo JY, Lim YY, Iyer P, Ong C, Tong JW, et al. Implementation of a nutrition screening tool to improve nutritional status of children with cancer in Singapore’s largest paediatric hospital. BMJ Open Qual. 2021 Mar;10(1).

175. Beckerleg W, Hasimja-Saraqini D, Kwok ESH, Hamdy N, Battram E, Wooller KR. Improving Timeliness of Internal Medicine Consults in the Emergency Department: A Quality Improvement Initiative. J Healthc Qual. 2020 Oct;42(5):294–302.

176. Wang Y, Zhang W, Gong X, Ong J, Marks M, Zhao P, et al. Optimizing Peer Distribution of Syphilis Self-Testing Among Men Who Have Sex with Men in China: A Multi-City Pragmatic Randomized Controlled Trial. Archives of Sexual Behavior. 2023 Jan 10;52.

177. Wu YP, Parsons BG, Nagelhout E, Haaland B, Jensen J, Zaugg K, et al. A four-group experiment to improve Western high school students’ sun protection behaviors. Transl Behav Med. 2019 May 16;9(3):468–79.

178. Gu Y, Bao X, Wang Y, Meng G, Wu H, Zhang Q, et al. Effects of self-monitoring devices on blood pressure in older adults with hypertension and diabetes: a randomised controlled trial. J Epidemiol Community Health. 2020 Feb;74(2):137–43.

179. Jiang Y, Ramachandran HJ, Teo JYC, Leong FL, Lim ST, Nguyen HD, et al. Effectiveness of a nurse-led smartphone-based self-management programme for people with poorly controlled type 2 diabetes: A randomized controlled trial. J Adv Nurs. 2022 Apr;78(4):1154–65.

180. Ramallo-Fariña Y, Rivero-Santana A, García-Pérez L, García-Bello MA, Wägner AM, Gonzalez-Pacheco H, et al. Patient-reported outcome measures for knowledge transfer and behaviour modification interventions in type 2 diabetes-the INDICA study: a multiarm cluster randomised controlled trial. BMJ Open. 2021 Dec 15;11(12):e050804.

181. Wan Y, Chen B, Li N, Yang JY, Dai HT, Tang KY, et al. Transradial versus Transfemoral Access for Patients with Liver Cancer Undergoing Hepatic Arterial Infusion Chemotherapy: Patient Experience and Procedural Complications. J Vasc Interv Radiol. 2022 Aug;33(8):956-963.e1.

182. Whinnett ZI, Sohaib SMA, Mason M, Duncan E, Tanner M, Lefroy D, et al. Multicenter Randomized Controlled Crossover Trial Comparing Hemodynamic Optimization Against Echocardiographic Optimization of AV and VV Delay of Cardiac Resynchronization Therapy: The BRAVO Trial. JACC Cardiovasc Imaging. 2019 Aug;12(8 Pt 1):1407–16.

183. Rooddehghan Z, Nezhad MM, Zakerimoghadam M, Karimi R. Effect of patient-centered and family-centered self-care education program on the quality of life of patients with multiple sclerosis: a quasi-experimental study. BMC Nurs. 2023 Oct 18;22(1):391.
